# Stress proteins: the biological functions in virus infection, present and challenges for target-based antiviral drug development

**DOI:** 10.1038/s41392-020-00233-4

**Published:** 2020-07-13

**Authors:** Qianya Wan, Dan Song, Huangcan Li, Ming-liang He

**Affiliations:** 1 Department of Biomedical Sciences, City University of Hong Kong, Kowloon, Hong Kong, China; 2 CityU Shenzhen Research Institute, Shenzhen, China

**Keywords:** Molecular biology, Drug regulation

## Abstract

Stress proteins (SPs) including heat-shock proteins (HSPs), RNA chaperones, and ER associated stress proteins are molecular chaperones essential for cellular homeostasis. The major functions of HSPs include chaperoning misfolded or unfolded polypeptides, protecting cells from toxic stress, and presenting immune and inflammatory cytokines. Regarded as a double-edged sword, HSPs also cooperate with numerous viruses and cancer cells to promote their survival. RNA chaperones are a group of heterogeneous nuclear ribonucleoproteins (hnRNPs), which are essential factors for manipulating both the functions and metabolisms of pre-mRNAs/hnRNAs transcribed by RNA polymerase II. hnRNPs involve in a large number of cellular processes, including chromatin remodelling, transcription regulation, RNP assembly and stabilization, RNA export, virus replication, histone-like nucleoid structuring, and even intracellular immunity. Dysregulation of stress proteins is associated with many human diseases including human cancer, cardiovascular diseases, neurodegenerative diseases (e.g., Parkinson’s diseases, Alzheimer disease), stroke and infectious diseases. In this review, we summarized the biologic function of stress proteins, and current progress on their mechanisms related to virus reproduction and diseases caused by virus infections. As SPs also attract a great interest as potential antiviral targets (e.g., COVID-19), we also discuss the present progress and challenges in this area of HSP-based drug development, as well as with compounds already under clinical evaluation.

## Overview of stress proteins

Stress proteins (SPs) are a diverse group of proteins that are synthesized at increased levels when cells are exposed to either intracellular or extracellular stressful stimuli. They exhibit protective effects against stresses. Stress proteins include heat shock proteins (HSPs), RNA chaperone protein (RNPs), and proteins mainly function in the endoplasmic reticulum (ER): peptidyl-propyl isomerases, protein disulfide isomerases (PDIs) and the lectin-binding chaperone system.^1^ SPs are ubiquitously expressed in all kind of cells, triggering signal cascades for neutralizing and eradicating the stresses occurring both intracellularly (e.g., pathogen invasion) and extracellularly (e.g., starvation, stimulation by cytokines/chemokines or hormones). Responses triggered by SPs can either activate pathways to promote cell survival or initiate cell death (i.e., apoptosis, necrosis, pyroptosis or autophagic cell death) for eliminating the damaged cells to protect a particular organ/tissue under given conditions. It is widely noted that the dysregulation of stress proteins is associated with a variety of human diseases, including cardiovascular diseases, neurodegenerative diseases (e.g., Parkinson’s diseases, Alzheimer disease), stroke, human cancers and infectious diseases. In this review, we focus on their functions and update findings involved in infectious diseases, particularly, the diseases caused by viral infections.

### Heat shock proteins

**Fig. 1:**
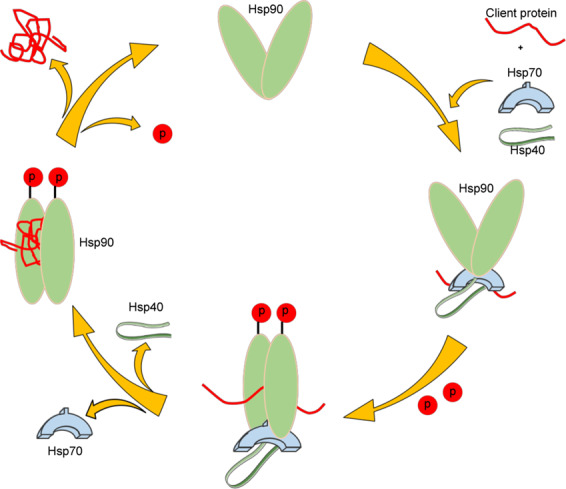
The general chaperone cycle of Heat shock proteins. Initially, unfolded client protein bound to the HSP70-HSP40 chaperones interacts with the HSP90. ATP binding to HSP90 induces the client proteins transfer from HSP70 to HSP90. Later, the conformation of HSP70-HSP40 chaperones will be released. Finally, the hydrolysis of ATP induces additional conformation changes leading to the client protein release

In 1962, an Italian geneticist Ritossa inadvertently elevated the incubation temperature of Drosophila larvae and discovered an increased gene transcription of unknown proteins. He nominated these protein as HSPs.^2^ Further studies have revealed a large number of HSPs, which form a big family and are ubiquitously expressed in cells. Based on the molecular weight, HSPs are classified into different families, including HSP100s, HSP90s, HSP70s, HSP60s, HSP40s, and some small HSPs (15–40 kDa).^3–5^ HSPs belong to the largest family of chaperones. The HSP expression is rapidly induced when cells meet physiological or environmental attacks such as starvation, high temperature, hypoxia or hyperoxia, pathogen invasion, malnutrition and exposure to chemicals or UV, etc. They form a network to promote or stabilize the correct folding of substrate protein to gain its functional/active conformation (Fig. 1), although they may not associate with the substrate protein in the final structure.^3,6^ HSPs are important factors in regulating cell survival, differentiation and cell death. Accumulating evidence shows that some HSPs participate in not only innate cellular immunity but also antigen presentation in adaptive immune response.^7,8^ HSPs also serve as potential biomarkers for some diseases. It has been shown that the increase of Hsp70 in plasma is associated with heart failure,^9^ and the elevated Hsp27 level in human peripheral blood mononuclear cells is related with coronary artery disease (CAD).^10^

#### Hsp90s

Hsp90, an abundant chaperone in all eukaryotic cells, controls a variety of critical signalling pathways in eukaryotic cells.^5,6,11^ Hsp90 is an ATP-dependent chaperone with different isoforms, including 1) Hsp90α (HSP90AA1, or HSPC1), Hsp90α-A2 (HSPAA2, or HSPC2) and Hsp90β (HSPAB1, or HSPC3) locate in cytoplasm. 2) **G**lucose **r**egulated **p**rotein Grp94 (HSPC4 or GP96) locates in the ER. 3) TRAP1 (HSPC5) locates in mitochondria.^12^ Among them, Hsp90α and Hsp90β account for the greatest proportion in humans. Hsp90 contains three regions: an ATPase-dependent hydrolytic domain in the N-terminal region, a middle linker region, and a dimerization domain in the C-terminal region.^5^ Like other HSPs, Hsp90 binds non-native substrate peptide to prevent its aggregation and degradation. When Hsp90 binds ATP, a transient dimerization of the N-terminal domain allows the substrate peptide binding to Hsp90. Then the ATP hydrolysis and energy release lead to a conformational change of the N-terminal domain that facilitates the correct folding of the substrate petite (Fig. 1).^5,6^ Besides, Hsp90 is also involved in telomere maintenance, apoptosis, and cell cycle progression, etc.^6,13^ It is well known that Hsp90 not only interacts and contributes to RNA polymerase assembly and nuclear import of some (−) ssRNA viruses (e.g., PB2 of influenza virus), but plays crucial roles in the folding process of viral capsid proteins and virion assemblies as well.^14^

#### *Cochaperones of Hsp90*

Cochaperones of Hsp90 regulate hsp90 functions at many aspects. CDC37 (also called p50) delivers kinase to Hsp90 and inhibits its ATPase activity; Carboxyl terminus of Hsp70-interacting protein (CHIP) functions as E3 ubiquitin ligase; Hsc70/Hsp90-organizing protein (HOP, also called STI1) inhibits dimerization of the N-terminal domain; and the activator of Hsp90 ATPase 1 (AHA) and p23 participates the maturation of substrate peptides.^11,15^

#### Hsp70s

HSP70 is a subfamily of HSPs’ superfamily with ~70 kDa molecular weight. It accounts for the majority of molecular chaperones in cells.^11^ The members of the Hsp70 family mainly include: (1) Hsp72 (HSPA1A), Hsp70-2 (HSPA2), Hsp70B’ (HSPA6) and Hsc70 (HSPA8) are commonly located in the cytosol; (2) Grp75 (HSPA9) is located in mitochondria; (3) Grp78 (HSPA5) is associated with the ER.^16^ Hsp70 consists of two domains: a 44-kDa nucleotide-binding domain (NBD) which can be divided into four subdomains (IA, IB, IIA, and IIB) in the N-terminal region and a 28-kDa substrate-binding domain (SBD) composed of C-terminal α-helical (SBDα) and N-terminal β-sheet (SBDβ) subdomains in the C-terminal region.^17,18^ As a critical component of cellular protein surveillance, the ATP-dependent molecular chaperone protects cells from damage caused by stress and takes part in a number of folding processes, including folding of newly synthesized polypeptides, recognition and refolding of misfolded or aggregated proteins, solubilization or degradation of proteins, transporting proteins, assembly or disassembly of oligomeric protein complexes, and the regulation of certain natively folded proteins.^5,13,19,20^

The functions of Hsp70 are not limited to host protein folding. Its functions are considerable during viral infection. The members of Hsp70s exhibit quite different roles in the course of virus life cycle. For example, Hsp72, Hsp70B’ and Hsc70 participate in the HCV viral entry, virion assembly and translation of the viral genome. Grp78 in ER is associated with the homeostasis of viral proteins and prevents the overload of viral proteins in host cells. In hepatocytes, the elevated Grp78 stimulates innate immunity to restrict or eliminate hepatitis B virus (B) replication.^21^ Grp75 interacts with the NS5A protein of HCV in mitochondria.^22^ Accumulating evidence shows that Hsp70 interacts with viral components of Human cytomegalovirus, Rabies virus, Respiratory syncytial virus, Human papillomavirus, Herpes simplex virus.

#### *Chaperone cycle of Hsp70*

The chaperone cycle is mediated by the N-terminal NBD, which regulates the binding of Hsp70 with substrates through switch of two states. The first state is the ATP- bound state with low affinity for substrate binding, i.e., a high association and dissociation rate of the substrate peptides to the SBD. The second state is ATP hydrolysis that switches to the ADP-bound and nucleotide-free state. At this state, the substrate exchange rates are low while the affinity to substrates is high. The chaperone activity of Hsp70 mostly relies on ATP hydrolysis. The basal ATPase of Hsp70 is normally low unless it is stimulated by the substrate peptide itself. It takes 20–30 min for a molecule of ATP to be hydrolysed per Hsp70 molecule at 30 °C. As a result, it is necessary for some cochaperones to encounter with Hsp70 ATP to induce ATP hydrolysis and help the increase of the affinity for substrate peptides.^19,23^

#### *Cochaperones of Hsp70*

The most crucial cochaperones of Hsp70 are members of the J- domain proteins (JDPs) family and nucleotide exchange factors (NEFs) family. Previous studies focused on the function of Hsp70 machinery and led to the development of a “canonical model” for its mode of action. The model contains two steps. First, the unfolded peptide substrates bind JDPs of the Hsp40 family; then the substrates are delivered to Hsp70 that stimulate the Hsp70’s ATPase activity. Simultaneously, JDPs prevent the aggregation of unfolded proteins. Second, NEFs work as substrate release factors that assure the substrate to fold into the correct and active conformation. In this way, the cochaperones strongly facilitate the function of Hsp70. Therefore, Hsp70 generally does not work individually but cooperates with cochaperones.^23,24^

#### Hsp60s

Hsp60s are large cylindrical oligomers with two back-to-back rings.^19^ The non-native proteins of the central cavity in each ring are folded into the native protein through an ATP- dependent process.^25,26^ Hsp60s are classified into two subfamilies. Group I is mainly in prokaryotes, while group II appears in eukaryotic cytosol and some archaea.^27,28^

The most well-studied one in Group I is the GroEL– GroES chaperonin system in the cytosol of bacteria. GroEL is an about 57 kDa protein with two rings arranged back-to-back; and 7 subunits form a tetradecamer structure. GroES is the cochaperone of GroEL.^11^ The two rings-tetradecamer structure appears in two forms include asymmetric (1 GroEL: 1 GroES) and symmetric (1 GroEL: 2 GroES) complexes, which are described as “bullet”^14,29,30^ and “American football”^31^ shaped respectively. The GroEL-GroES chaperonin undergoes allosteric regulation dependent on ATP and which completes the protein folding function. The polypeptide binds to the hydrophobic sites of one of the seven subunits of GroEL and changes conformation upon ATP binging and hydrolysis. With the help of cochaperone GroES, the polypeptide finishes its folding process.^32^ In contrast to the GroEL–GroES system, the mammalian homolog Hsp60/Hsp10 system is less studied. Hsp60 is thought to be imported into the mitochondria and converted into its mature form with a molecular size of 58 kDa.^33–35^ Hsp60 exhibits important roles in facilitating protein folding, transportation and proteostasis in mitochondria.^36,37^

Group II chaperonins include the archaeal thermosome and eukaryotic CCT (chaperonin-containing TCP1, or called TriC), which are oligomers with eight to nine subunits with molecular weight 57–61 kDa in each ring. Compared to group I, group II chaperonins show different allosteric movements by ATP binding.^11,19^

HSP60 family is known to participate in viral life cycle at various stages from viral attachment to the replication of the viral genome. Hsp60s are essential for host cell immunity regulation. Some viral proteins require Hsp60 for its translocation within host cells. PB2 is a subunit of Influenza A viral RNA polymerase, which mostly locates in the nucleus but also appears in mitochondria.^38^ When the virus infects the host cells, PB2 is responsible for maintaining the function of mitochondria. PB2 interacts with mitochondrial antiviral signaling protein (MAVS) to downregulate intracellular immune response by decreasing the level of IFNβ so that the invading virus can easily escape from the defence of host cells.^39^ Hsp60 takes the role of transporting PB2 from cytosol to mitochondria in the host cells. Besides, Hsp60 shows great regulatory functions on innate immunity by inducing pro-inflammatory cytokine release, such as TNF-a, IL-6, and IL-1b, etc.^40^

#### Small heat shock proteins

Small heat shock proteins (sHSPs) are a group of small proteins with a low molecular weight ranging from ~15 to 40 kDa.^4^ There are 10 members in the sHSP family and some are ubiquitous including Hsp27 (HSPB1), HSPB5 (αB-crystallin, or αBC), Hsp20 (HSPB6), and Hsp22 (HSPB8), while the others are tissue-specific including HSPB2 (myotonic dystrophy protein kinase, or MKBP), HSPB3, HSPB4 (αA-crystallin, or αAC), HSPB7 (cvHsp), HSPB9, and HSPB10 (sperm outer dense fiber protein, or ODF).^41^ sHSPs can exist as monomers, dimers or even large multimeric complexes in the cells.^42^ The structure of sHSPs is different from the other HSPs due to their less conserved sequences. The basic structure of sHSPs is a conserved α-crystallin domain (ACD) flanked by two non-conserved domains including the N-terminal sequence (NTS) and the C-terminal sequence (CTS). Among these domains, ACD becomes the characteristic of different sHSPs.^43,44^

sHSPs play crucial roles in several physiological processes regarding stress tolerance, apoptosis, aging, and longevity.^45–48^ The phosphorylation together with the N-terminal WDPF motif helps sHSPs to form homo- or hetero-oligomers.^49,50^ The oligomerization is the hallmark of sHSPs for supporting their quite different activities. Phosphorylation favors small oligomer formation while dephosphorylation provokes a shift toward large oligomer formation.^51^ Oligomerization dynamics is crucial for chaperone activity because it gives rise to the possibility to format different homo- and hetero-oligomers, each with different binding properties to chaperone a broad range of substrates.^41,52^ For instance, the phosphorylated species are required for actin dynamics. Small phosphorylated dimers/tetramers bind F-actin to regulate actin polymerization.^53^

Among different sHSPs, Hsp27 has been broadly studied. Hsp27 exists in all tissues though it is known to mainly express in cardiac, skeletal and smooth muscles.^54^ Its importance has been demonstrated in cell differentiation, cell survival, cellular innate immunity, viral protein translation, and intracellular virus transport, etc.^55–57^ Same as the other sHSPs, Hsp27 shares a highly conserved α-crystallin domain.^41,58,59^ Hsp27 is capable of oligomerization and phosphorylation. There are three serine residues 15, 78, and 82 can be phosphorylated by different kinases including p90Rsk, PKG, MAPKAP kinases, etc.^55^ The phosphorylation of Hsp27 is a reversible process. The dephosphorylation contributes to the formation of large size oligomers.^60,61^ Hsp27 can not only form large homo oligomers up to 800 kDa; but it can also cooperate with other sHSPs (e.g., Hsp20) to form heteromeric structures.^56,58^ Hsp27 is upregulated and activated upon infections.^62,63^ The elevated Hsp27 activity promotes either cytoskeletal stability or cell motility,^64,65^ and prevents apoptosis.^66^ Hsp27 is required for IL-1- induced expression of the pro-inflammatory mediators IL-6, IL-8, and cyclooxygenase-2.^67–69^ Hsp27 is also linked to various signalling pathways involved in the development, differentiation, and cell growth.^70,71^ The long-term and high-level expression of Hsp27 stimulated by variety of stresses (such as HBV or EBV infections) enhances carcinogenesis, cell survival, stemness of cancer cells, cancer metastasis, tumour formation and drug resistance.

#### Transcriptional regulation of the HSPs

Heat shock factors (HSFs) display great contributions in regulating the transcriptional activation of *hsp* genes. In all invertebrate animals, only HSF1 is responsible for the transcriptional activation. In vertebrates, four members of HSF family (HSF1-4) regulate HSP expression.^72^ Among them, HSF1 is the most critical one. The fibroblasts from hsf1^−/−^ mice undergo apoptosis upon heat stress because of no *hsp* transcription.^73^ Upon stress conditions, the originally monomeric HSF1 in the cytoplasm could trimerize and translocate into the nuclei to promote the *hsp* expression by binding on the heat shock elements (HSE) in the promoter region.^74^

### Protein disulfide isomerase

Protein disulfide isomerase (PDI) is a multifunctional oxidoreductase and chaperone that catalyses the formation, isomerization and reduction of disulfide bonds in the endoplasmic reticulum (ER). During disulfide bond formation, cysteine residues at the CGHC active site of PDI accept two electrons from the cysteine residues in polypeptide substrates, leading to the reduction of PDI and oxidation of the substrate. Then PDI transfers the electrons to an acceptor to start another cycle of disulfide bond formation.^75^ In addition to PDI’s catalytic function as a thiol-disulfide isomerase, it also exhibits molecular chaperone properties for glycosylated protein quality control.^76^ ERp57 (PDIA3, Grp58) is possibly the most thoroughly studied PDI family member that shares a similar structure consisting of four domains (namely a-b-b’-a’) and possesses two localization sequence—an ER retention signal (QDEL), and a nuclear localization signal (KPKKKKK). Unlike other PDI family members that directly bind the substrates for their reductase or isomerase activities, the b domains of ERp57 have a high affinity to associate with calreticulin (CRT) and calnexin (CNX), which would help to recognize and recruit polypeptide segments of the glycoproteins.^77^ If the protein is not correctly folded, UDP-glucose:glycoprotein glucosyltransferase (UGGT) would be recruited to reglycosylate the proteins, allowing them to be recognized and re-associated by ERp57/CRT/CNX complex.^76,78,79^ Considering the essential roles of PDIs in the oxidative folding and chaperone-mediated protein quality control, they are now linked to a growing range of diseases including those are caused by virus infection.

### RNA chaperones

Proteins that interact non-specifically with RNA and resolve the non-functional inhibitory structures are usually referred to as RNA chaperones, which have distinct roles without common sequences or motifs.^80,81^ They participate in a large number of cellular processes, including chromatin remodelling, transcription regulation, RNP assembly and stabilization, RNA export, histone-like nucleoid structuring, intracellular immunity, and viral RNA replication and translation. RNA molecules mostly rely on well-defined 3D structures to fulfill their functions. However, the process of RNA folding is very complicated.^82^ The multitude of possible RNA base-pairings together with the high stability of RNA duplexes would give rise to a large number of alternative secondary and tertiary structures that are thermodynamically as stable as the functional, native structure.^83^ RNA chaperones promote RNA folding by accelerating the escape from kinetic folding traps and prevent RNAs from being trapped in non-functional conformations.^84–86^ So far, no protein has been characterized whose primary function is to resolve non-specifically misfolded RNAs in cells.^80,81^

HnRNPs are a group of heterogeneous nuclear ribonucleoproteins. They are essential factors for manipulating both the functions and metabolisms of pre-mRNAs/hnRNAs transcribed by RNA polymerase II. More than 20 hnRNPs have been identified to date. hnRNPs contain common RNA binding motifs like arginine glycine boxes (RGG boxes), RNA recognition motifs (RRMs), hnRNP K homology (KH)-domains and zinc finger (ZF)-domains (KHZF domain).^87^ Well-defined functions of this family include transcription regulation, pre-mRNA splicing, 3′-end formation, mRNA packaging, RNA transport, translational regulation, RNA silencing, DNA repair, and telomere biogenesis. They also have the ability to shuttle between nucleus and cytoplasm, therefore could transiently help to form RNP complexes in nucleus and also participate in RNA metabolism in cytoplasm.^88^ A large collection of hnRNPs are involved in virus activities, most of which were first identified using viral RNA–protein binding assays, followed by functional assays.^89^

### The importance of stress proteins

**Fig. 2:**
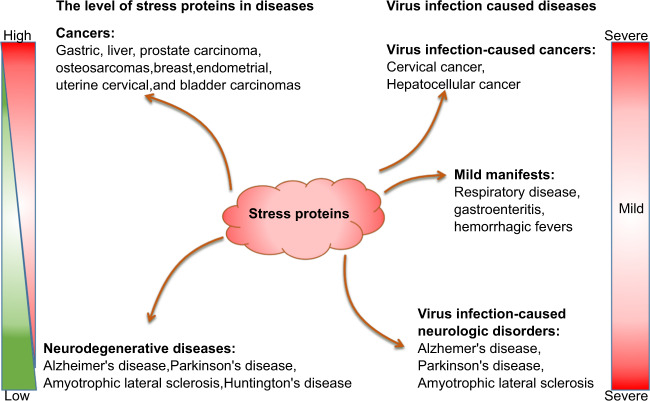
Diseases caused by dysfunction of stress proteins and their dysregulation by virus infections. The left panel shows the expression level of stress proteins in diverse human diseases, including cancers and neurodegenerative diseases. The expression level of stress proteins is significantly elevated in cancer cells but dramatically decreased in neurodegenerative disease. The right panel shows the virus infection-caused diseases from mild to severe which include cancers and neurodegenerative diseases. Stress proteins contribute to these diseases

One of the main functions of stress proteins is to maintain cellular homeostasis. Under pressure, stress proteins are hyperactive to release the pressure. Hsp27, Hsp70 and hsp90 accumulate to a very high level in quite a few types of cancer cells.^90–92^ Although the mechanism underlying the increase has not been fully understood, it suggests that the fast increased HSPs respond to the folding stresses. With tumor progression, the accumulating oncogenic proteins need powerful protein folding ability. Under long-term stresses, HSPs participate or promote carcinogenesis, cell survival, anti-apoptosis, angiogenesis, cancer cell stemness, invasion and metastasis.^93^ However, HSPs and RNA chaperones are downregulated in nearly all age-related neurodegenerative diseases including Alzheimer’s disease, Parkinson’s disease, amyotrophic lateral sclerosis, and several polyglutamine diseases such as Huntington’s disease and different forms of spinocerebellar ataxias^94,95^ (Fig. 2). Downregulated RNA chaperones lead to disorder of RNA metabolism;^94^ while the attenuated HSPs result in a failure of the protein quality control (PQC) system to adequately handle the folding or timely degradation of proteins in neurologic disease.^95^ Both mechanisms cause protein aggregates, the hallmark of age-related neurodegenerative diseases. In this review, instead of paying much attention to these topics, we would only focus on the main biological functions and target values of stress proteins in human diseases caused by pathogen infections, particularly by virus infections because the well-developed antibiotics have already controlled the bacterial infection very well since 1950s.

Viruses infection causes a variety of diseases that are highly associated with dysregulation of stress proteins (Fig. 2), e.g., respiratory symptoms,^96,97^ gastroenteritis,^98–101^ Hemorrhagic fevers.^102,103^ Critically, it has been demonstrated that neurodegenerative diseases as well as neurobehavioral disorders are the consequences of loss of neurons and axons in the central nervous system with ageing. It is evidenced that these diseases are possibly caused by chronic neuropathic viral infections.^104^ For example, HIV infection can cause neurocognitive disorder. To date, increasing evidence shows that systemic viral infections are often associated with some neurodegenerative diseases, including Alzheimer’s disease, Parkinson’s disease, amyotrophic lateral sclerosis, multiple sclerosis, autism spectrum disorders.^104,105^ Over 20% of cancer cases are attributable to viral infection worldwide. The viruses associated with the greatest number of cancer cases are the human papillomaviruses (HPVs) and hepatitis viruses (HBV and HCV). HPV infection causes cervical cancer and several other epithelial malignancies, and HBV/HCV infections are responsible for the majority of hepatocellular carcinoma. Other oncoviruses include Epstein-Barr virus (EBV), Kaposi’s sarcoma-associated Herpes virus (KSHV), human T-cell leukemia virus (HTLV-I), and Merkel cell polyomavirus (MCPyV), and HIV.^106^ Regarding these diseases caused by the virus infections, a lot of fundamental questions have not been fully addressed. In this review, we pay special attention to the impact of stress proteins responding to virus infections on both virus reproduction and pathogenesis of diseases.

**Fig. 3:**
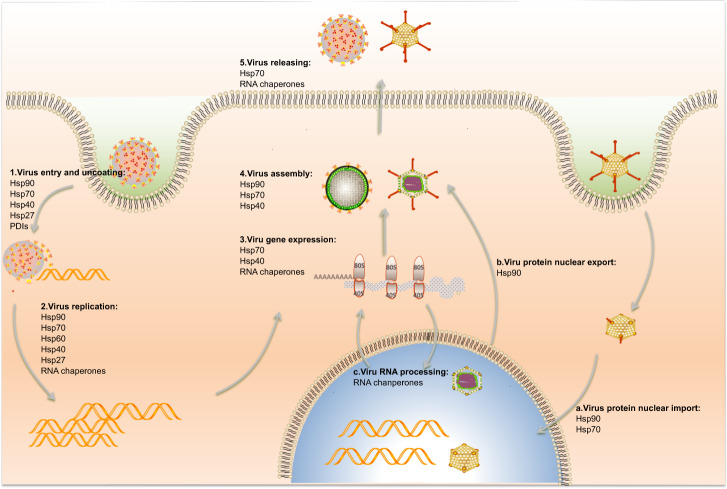
Stress proteins participate in diverse steps of the virus life cycle. Five major steps of the virus life cycle are labeled as steps 1–5: virus entry and uncoating, virus replication, gene expression, assembly and release. Most viruses only undergo these steps in cytoplasm especially RNA viruses like EV-A71, DENV, JEV etc. While some viruses (such as influenza, SV40, HBV etc) also enter into nucleus of host cells. They may undergo other steps in their life cycle which are labelled as a–c: virus nucleus import, nucleus export, and virus RNA processing. During the process of virus infection, Hsp90, Hsp70, Hsp60, Hsp40, Hsp27, and PDIs participate in the virus entry and uncoating steps. Hsp90, Hsp70, Hsp60, Hsp40, Hsp27 and RNA chaperones take part in the virus replication step. Hsp70, Hsp40 and RNA chaperones are required in virus gene expression step. Hsp90, Hsp70 and Hsp40 assist virus assembly. Hsp70 and RNA chaperones contribute to the virus release. While Hsp90 is also important in virus nucleus import and export. Hsp70 plays a role in the virus nucleus import. And RNA chaperones play a major role in virus RNA processing, including replication, initiation of translation, stabilization and decay

Stress proteins are involved in many steps of virus infection process, including virus entry, uncoating, replication, gene expression, as well as virus assembly and releasing as steps 1–5 shown (Fig. 3) Some viruses replicate in the nucleus, while stress proteins take part in the virus protein and/or RNA nuclear import/export processes as setp a–c shown (Fig. 3). The summary of the relationship between stress proteins, virus infection as well as host cell response is listed in Table 1.

**Table 1:** Stress proteins participate in different steps of diverse viruses replication processes

Chaperone family	Selected Members	Function in viral infection	Related viruses
Hsp90		Virus entry	RNA virus: EV-A71,^107^ DENV,^110^ JEV^108^
			DNA virus: HSV,^131^ HBV^132^
		Virus replication	RNA virus: Influenza,^118^ Paramyxoviruses:^112,113^ VSV, HPIV-2, HPIV-3, SV41 or RSV; CHIKV,^114^ HCV^115^
			DNA virus: HSV,^144,145^ EBV,^147,163^ HBV^135^
		Virus Protein Maturation and Virus Assembly	RNA virus: HCV,^123^ Picornaviruses, Poliovirus, Rhinovirus and Coxsackievirus,^124^ Noroviruses,^125^ Influenza^126^
			DNA virus: HBV^169,170^
		Virus gene expression	DNA virus: HSV,^150^ HCMV,^151,152^ VZV, EBV, KSHV,^153^ EBV^146,164^
		Virus-induced Tumorigenesis	DNA virus: EBV^175,176^
		Immunity modulation	DNA virus: EBV^177^
		Cellular transformation	RT virus: HTLV^153^
Hsp70	GRP78 Hsc70 HSP70 HSP72	Virus entry	RNA virus: CAV-9,^186^ EV-A71,^188^ DENV,^110,189^ JEV,^191^ ZIKV^190,197^
			DNA virus: SV40,^234^ AdV,^237^ HSV,^241^ polyomavirus^254^
			RT virus: HTLV-1,^258,259^ HIV-1^260^
		Virus replication	RNA virus: MuV,^201^ CDV,^203,204^ HCV,^206^ RSV,^209^ EBOV,^212,213^ Influenza^217^
		Virus gene expression	RNA virus: Coxsackievirus B3,^218^ EV-A71,^219^ Influenza A^222–226^
			DNA virus: Polyomavirus, JCV, HCMV,^245,246^ HBV^247–249^
		Assembly	RNA virus: Reovirus,^227^ Poliovirus,^228^ Coxsackievirus B1,^228^ Influenza^229^
			DNA virus: Polyomavirus^254^
		Virus release	RNA virus: HCV^232,233^
		Cellular transformation	DNA virus: SV40,^264–267^ HPV,^275^ AdV^278^
		Cell survival and apoptosis	DNA virus: EBV^282^
		Immunity modulation	DNA virus: HBV^294^
Hsp60		Virus replication	DNA virus: HBV^325,326^
		Immunity modulation	RNA virus: JEV,^317^ Influenza,^38,39,318^ DENV^319^
			DNA virus: HBV^331,335^
		Apoptosis regulation	RNA virus: HCV,^320–322^ Rotavirus^323^
			DNA virus: HBV^329,330^
		Genome integration	RT virus: HIV^336,338^
Hsp40	Hdj2 Hsp40/DnaJB1 Hsp40/DnaJA1 Hsp40/p58IPK DNAJC14 DNAJA3 Hdj1 hTid1 DnaJB6	Virus entry	RT virus: HIV^374,375^
		Virus replication	RNA virus: JEV,^339^ Influenza^340,343,344^
			DNA virus: HPV,^355–357^ HSV,^359,360^ HBV ^361,362^
		Virus Gene expression	RNA virus: Influenza^225,346^
			RT virus: HIV^377–379^
		Protein maturation	RNA virus: YFV^350^
		Immunity modulation	RNA virus: HFDV^354^
		Cellular transformation	DNA virus: HBV,^363–366^ HPV^275^
Small chaperone	Hsp27	Viral replication	RNA virus: EV-A71,^384^ Swine fever virus (CSFV),^388^ Porcine epidemic diarrhoea virus (PEDV)^389^
			DNA virus: Adenovirus,^390^ HSV-1,^397^ RRV,^397^ PCV2,^399^ EBV^386^
PDIs	PDI ERdJ5 ERp57	Virus entry and uncoating	RNA virus: Newcastle disease Virus,^402^ DEGV^402–404^
			DNA virus: SV40^413^
			RT virus: HIV^407,410^
		Virus translation	RNA virus: EV-A71^415^
		Oxidative stress and ER stress	RNA virus: Influenza,^419^ HCV,^421^ EMCV,^422^ RSV,^423^ JEV^424^
			DNA virus: HBV,^420^ HPV^425^
			RT virus: HIV^418^
RNA chaperone	hnRNPA1 hnRNPA2 hnRNP A/B hnRNP A2/B1 hnRNPC hnRNPD hnRNPE hnRNPI/PTB hnRNPH hnRNPK hnRNPL hnRNPM	Virus replication	RNA virus: JEV,^435^ Coronavirus,^436^ Poliovirus,^438^ HCV^437^
			DNA virus: HCMV^488^
		RNA transcription	DNA virus: HPV18,^425,489^ HBV,^490^HSV^492^
			RT virus: HIV^514^
		RNA polyadenylation	RT virus: Rous sarcoma virus^485,486^
			DNA virus: HPV,^497,498^ EBV^501^
		Virus RNA splicing	RNA virus: Influenza A virus (IAV)^449^
			DNA virus: HPV^502^
			RT virus: HIV^529^
		Virus RNA export	RNA virus: Influenza^484^
			RT virus: HIV,^556^ Kaposi’s sarcoma-associated herpesvirus (KSHV),^560,561^ HTLV-1^562^
		Virus translation	RNA virus: HCV,^452–454^ HRV-2,^89^ EMCV,^456^ calicivirus,^457^ EV-A71,^122,471^ Coxsackievirus B3(CVB3),^474^ NiV, DENV^476^
			DNA virus: HPV18^508^
			RT virus: HIV,^563^ KSHV^564^
		Cellular transformation	DNA virus: EBV^510^
		Immunity modulation	DNA virus: HSV^512^

## The function of HSP90 family in virus infection

In this section, we would review and present new findings on HSP90 family proteins in the life cycles of different viruses including RNA virus, DNA virus and retrovirus. In addition, we also discuss their functions in virus-induced cellular response.

### The function of HSP90 family in RNA virus infection

#### Virus entry

In the case of RNA virus, Hsp90 is critical for the entry of enterovirus A71 (EV-A71),^107^ Japanese encephalitis virus (JEV) and Dengue virus (DENV).^108^ EV-A71 entry is significantly blocked when cells are pre-treated with Hsp90 inhibitors or Hsp90-specific siRNAs.^107,109^ Since both DENV virus and JEV belong to the flavivirus, the entry of the these two viruses differently utilize Hsp90 with the support of Hsp70s in both neuroblastoma and microglial cells.^95,108^ DENV depends much more on Hsp90 to enter into cells as compared with JEV since anti-Hsp90 antibodies or Hsp90 inhibitors block the majority of DENV entry^110^ but only a small portion of JEV entry.^111^ Additionally, both Hsp90 and Hsp70 are associated with membrane lipid rafts in response to DENV infection.^110^ However, in DENV infected liver cells (HepG2), neither Hsp90 nor Hsp70 works as the receptor to enable DENV internalization,^110^ indicating alternative entry mechanisms in different cell types. It is likely that the receptor functions of Hsp90 and Hsp70 are replaced by other unknow molecules.

#### Virus replication

Hsp90 protein facilitates virus replication in several aspects. First, Hsp90 works as a classic chaperone protein to stabilize virus proteins. Hsp90 stabilizes paramyxoviruses polymerase and L protein, as well as assists virus replication. Inhibition of Hsp90 could hamper virus replication and shorten the half-life of L protein in vesicular stomatitis virus (VSV), human parainfluenza viruses-2 (HPIV-2), human parainfluenza viruses-3 (HPIV-3), simian virus 41 (SV41), or respiratory syncytial virus (RSV) infection cells.^112,113^ Similarly, Hsp90 is shown to maintain the stability of chikungunya virus (CHIKV) non-structural proteins (nsPs) including nsP3 (a protein essential for RNA synthesis) and nsP4 (RNA-dependent RNA polymerase, RdRp);^114^ and the protease of HCV nonstructural protein NS3.^115^ Second, Hsp90 modulates virus polymerase activity to enhance virus replication. Taking HCV as an example, Hsp90 indirectly modulates the HCV polymerase NS5 activity by maintaining the stability of kinase phosphoinositide dependent kinase l (PDK1), an upstream kinase of NS5 phosphorylation kinase PRK2.^116^ Besides, mediated by FKBP8, Hsp90 forms a complex with NS5 and directly regulates NS5 activity.^117^ Hsp90 inhibitors suppress viral replication by disrupting the Hsp90-NS5 complex formation.^117^ Third, Hsp90 manipulates the proper location of virus polymerases. During influenza virus infection, Hsp90 interacts with the viral RdRp subunits polymerase basic protein-1 (PB1) and -2 (PB2) to form a complex, and then co-translocates into nucleus.^14,118^ In this process, Hsp90 maintains the stability of PB1 and PB2. After entry into nucleus, Hsp90 dissociates from the Hsp90/PB1/PB2 complex and forms a new functional complex with polymerase acidic protein (PA).^119^ Extending study shows that the state of Hsp90 acetylation is strictly regulated by histone deacetylases 6/8 (HDAC6/8) in the influenza RdRp nuclear import stage. HDAC6/8 inhibitors efficiently limit the polymerase nuclear import and suppress virus replication.^120^ In the course of influenza infections, Hsp90 expression is stimulated through mTOR/p70S6K signalling.^121^

**Fig. 4:**
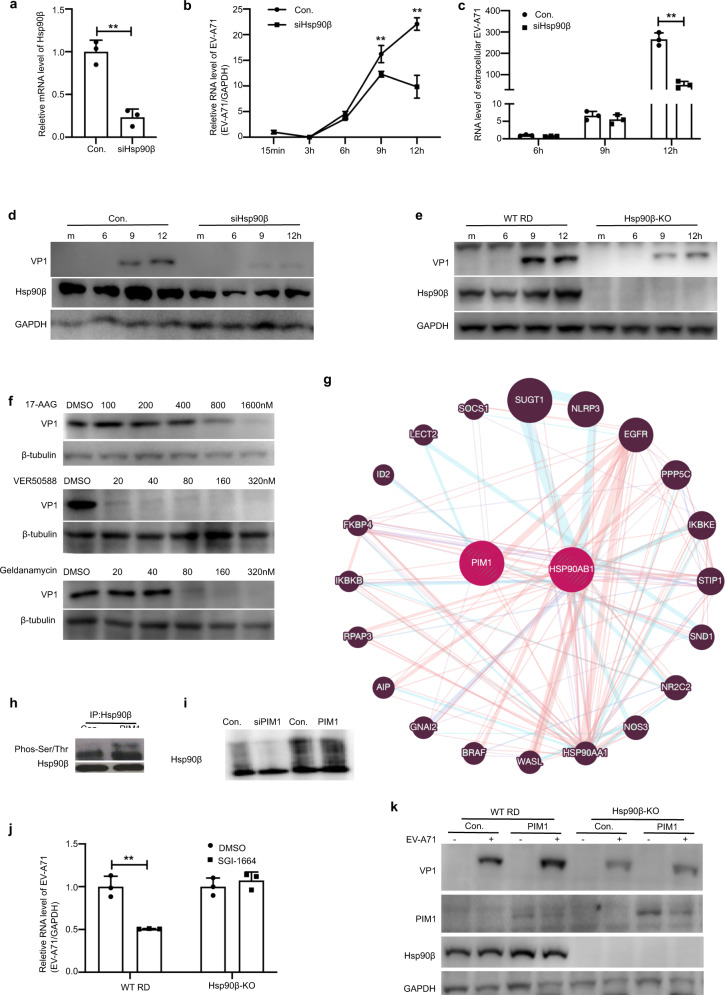
Pim1 signaling promotes EV-A71 replication through Hsp90β phosphorylation(unpublished data). **a** RD cells were treated with scramble/Hsp90β siRNA for 24h, the effects of Hsp90β knockdown were determined by RT-qPCR assay. The results are shown as the means, and ±error indicates the standard deviation (SD). Data are obtained from triplicate experiments. *p < 0.05 and **p < 0.01 by two-tailed Student’s *t*-test. **b**, **c** RD cells were treated with scramble /Hsp90β siRNA for 4h, then infected with EV-A71 at MOI of 1 for indicated time. The intracellular (**b**) and extracellular (**c**) viral RNA levels were detected by RT-qPCR assay. The results are shown as the means, and ±error indicates the standard deviation (SD). Data are obtained from triplicate experiments. *p < 0.05 and **p < 0.01 by two-tailed Student’s *t*-test. **d**, **e** knockdown of Hsp90β by siRNA or knockout by CRIPSR/Cas9 mediated gene editing in RD cells, and then cells were infected with EV-A71 at MOI of 1 for indicated time. The protein level of EV-A71 was determined by western blot. **f** RD cells were treated with Hsp90 inhibitors at different concentrations and infected with EV-A71 at MOI of 0.01 for 48h. The protein level of EV-A71 was determined by western blot. **g** Pim1-protein interaction network was predicted using online *GENEMANIA* program. **h** Pim 1 was overexpressed in 293T cells and cell lysate was collected for immunoprecipitation assay. The phosphorylation status of Hsp90 was detected by western blot. **i** Pim1 was overexpressed/knocked down in 293T cells. And cell lysate was collected for native page analysis. **j** WT RD cells and Hsp90β knockout (Hsp90β-KO) cells were pretreated with 2 μM PIM1 inhibitor SGI-1776 for 2 h, then the cells were infected cells with EV-A71 at MOI of 0.01 for 48h. Intracellular viral RNA level was determined by RT-qPCR. The results are shown as the means, and ±error indicates the standard deviation (SD). Data are obtained from triplicate experiments. *p < 0.05 and **p < 0.01 by two-tailed Student’s *t*-test. **k** Pim1 was overexpressed in WT/Hsp90β -knockout RD cells, and infected with EV-A71 at MOI of 1 for 12 h. The protein level of EV-A71 was determined by western blot

Our recent studies show that Hsp90 also exhibits significant importance on EV-A71 replication through PIM1 signalling (unpublished). It has been shown that EV-A71 infection elevated both the mRNA and protein levels of PIM1.^122^ The elevated PIM promoted EV-A71 replication while knockdown of PIM1 decreased EV-A71 replication. Knockdown of Hsp90β decreases 60% of virus replication 12h post infection (p.i.), while the secreted virions decrease by approximately 80%, indicating the crucial roles of Hsp90β in both virus replication and secretion (Fig. 4a–c). Other researchers reported that Hsp90β is responsible for EV-A71 assembly which may be the reason that Hsp90β attenuates the secretion of EV-A71 virions in our study.^107,109,122^ Notably, Hsp90β contributes to virus replication more than that of secretion. Our data also shows that knockdown or knockout of Hsp90β decreased the proteins expression level of EV-A71 structure protein (Fig. 4d, e). And Hsp90 inhibitors 17-AAG, Geldanamycin (GA) and VER50588 all dramatically inhibit EV-A71 protein expression (Fig. 4f). Among them, VER50588 display the strongest inhibitory effect which has not been reported before. We predicted that Hsp90β is a potential target of PIM1 (Fig. 4g). To address this hypothesis, we conducted experiments by overexpression PIM1 and knockdown of PIM1. Accordingly, the phosphorylated status of Hsp90β is increased and decreased (Fig. 4h,i). More importantly, knockout of Hsp90β by CRISPR/Cas9-mediated gene editing almost completely abolishes the effects of PIM1 signaling on EV-A71 replication (Fig. 4j, k).

#### Viral protein maturation, virion assembly, and release

During the viral protein expression and maturation, Hsp90 works as a classic chaperone to monitor the proper folding of viral proteins. Hsp90 modulates the maturation of HCV nonstructural protein 2/3 (NS2/3) kinase.^123^ HCV NS2/3 is cleaved into two separate proteins right after translation, a key step of NS2/3 protein maturation. Hsp90 strictly regulates the proper folding of newly synthesized NS2/3 protein.^123^ Hsp90 and its co-chaperone p23 form a complex to assist the proper folding of capsid precursor polyprotein P1 of poliovirus, rhinovirus, and coxsackievirus;^124^ while the inhibitor GA reduces the maturation of P1, leading to immature P1 degradation in proteasome.^124^ During the virion assembly, Hsp90 interacts with capsid VP1 protein of noroviruses and the termini of the murine norovirus 1 genome.^124,125^ This interaction not only stabilizes VP1, but ensures the viral genome to be encapsulated into capsids as well.^124^ Hsp90 interacts with and stabilizes influenza neuraminidase (NA), a major surface glycoprotein involving in virion release.^126^ More importantly, it emphasizes the Hsp90-NA complex formation on promoting cell survival, leading to more virus production.^126^

### The function of HSP90 family in DNA virus infection

#### Virus entry

The entry of DNA viruses includes steps of crossing over cell membrane and nuclear import. Hsp90 is mainly shown the ability to assist the nuclear import of virus. The nuclear transport of many viruses depends on the microtubules (MT) and MT-dependent molecular motor dynein/dynactin complex.^127^ Virus strictly modulates the status of tubulin acetylation, a critical event for the transportation of viral components.^128–130^ In HSV-infected cells, Hsp90 co-localizes with acetylated tubulin and capsid protein VP5.^131^ Hsp90 inhibitors disrupt its binding to the acetylated tubulin, thereby inhibiting the nuclear transport of HSV capsid protein.^131^ During HBV infection, the glucocorticoid receptor shows a strong possibility to enhance the nuclear import of HBV.^132^ Hsp90 facilitates glucocorticoid receptor redistribution from the cytoplasm to the nucleus.^133^

#### Virus replication

During DNA retroviral replication, Hsp90 mainly contributes to regulating and maintaining the reverse-transcriptase (RT) activity. Taking hepatitis B virus (HBV) as an example, the reverse transcription is an essential step to generate viral genomic DNA in type VII viruses. The beginning of reverse transcription is the recognition and interaction of RT with an RNA signal (the packaging signal, ε) on the pre-genomic RNA.^134^ It was identified that Hsp90 is an essential host factor that facilitates Duck hepatitis B virus (DHBV) replication by interacting with viral RT.^135^ Treating with Hsp90 inhibitors or monoclonal antibodies (mAb) sufficiently block RT-ε binding.^135^ Hu et al. demonstrated that RT-ε interaction depends on Hsp90’s ATP hydrolysis activity.^136^ Two independent regions in the terminal protein (TP) and the RT domains of polymerase separately bind with Hsp90 at the N-Terminal and C-Terminal fragments, and both domains are essential for ribonucleoprotein (RNP) and protein priming.^137,138^ Although a model is established to show how Hsp90 bridges the two separate RT domains of polymerase together to enable the formation of an RNP complex with the HBV RNA;^137^ there are still some fundamental questions to be addressed. Firstly, whether the Hsp90 chaperones or Hsp70 chaperones are essential for the RT- ε interaction. Stahl et al. believed that Hsp70 chaperones are much more important for the RT–ε interaction. They proposed that Hsp90/Hop complex affect the quantity, not the quality of RT activity.^139^ While Hu et al. stated that Hsp90 is critical for the RT activity.^137,140,141^ Secondly, Stahl et al. believed that the stimulated RT activity is independent of Hsp90 ATPase activity;^139^ while Hu et al. reported that RT-ε interaction mediated by Hsp90 and its chaperone partner p23 is ATP-dependent.^137,141^ Lastly, another study showed that Hsp90 helps HBV RT priming rather than maintaining the HBV RT/ε RNA complex.^142^ This is controversial to Hu’s findings, i.e., Hsp90 function is required not only to establish but also to maintain the RT in a state for RNA binding.^141^ Grp94, another HSP90 family member, is shown a critical regulator in stabilizing and activating RT, allowing its preferential binding to the pregenomic RNA during HBV replication.^143^

The replication of most DNA viruses occurs in the nucleus, where virions form in replication centres. Therefore, the proper location of viral proteins is quite important for virus replication. Hsp90 is also found to regulate the location of virus DNA polymerase in virus-infected cells. After treated with Hsp90 inhibitors, HSV polymerases is mislocalized from the nucleus to the cytoplasm and subsequently degraded in a proteasome-dependent manner.^144,145^ Similar to HSV, the nuclear translocation of DNA polymerase of EBV also requires Hsp90.^146,147^ During the polymerase transportation, the polymerase catalytic subunit BALF5 forms a complex with BMRF1 in the assistance of Hsp90β. Hsp90 inhibitors effectively block the translocation of viral DNA polymerase.^146,147^

#### Virus gene expression

Hsp90 is important for virus gene expression both at the transcription and translation levels. The transcription of HSV immediate-early α (IEα) genes is initiated by the transcription factor complex, which is composed of octamer-binding transcription factor 1 (Oct-1), host cell factor 1 (HCF-1) and viral protein 16 (VP16).^148,149^ In the complex, VP16 is the major virus-encoded transcriptional activator that controls the efficiency and level of viral gene transcription. In the transcription process, Hsp90α is shown to maintain the stability of VP16 by keeping it from degradation in a macroautophagy-mediated manner.^150^ Similarly, Hsp90 also regulates the transcription of human cytomegalovirus (HCMV) immediate-early genes through activating Akt and NF-κB signalling pathways, which are critical for major immediate early genes transcription.^151,152^

At the translation level, Hsp90 promotes the translation of conserved herpesvirus protein kinases (CHPKs), including herpes simplex virus type 1 and 2 (HSV-1, HSV-2), varicella-zoster virus (VZV), EBV, KSHV.^153^ CHPKs play important roles in multiple processes, including gene expression,^154–156^ viral DNA replication,^156–158^ capsid nuclear egress,^159,160^ and DNA damage responses.^161,162^ The translation of EBV nuclear antigen 1 (EBNA1) protein is also manipulated by Hsp90.^163,164^ EBNA1 is critical for cellular transformation, tumorigenesis, and the maintenance of viral episomes.^165–167^ The EBNA1 translation is strictly regulated by Hsp90 through the Gly-Ala repeat domain to keep EBNA1 at a relatively low level.^168^ It has been demonstrated that Hsp90 inhibitors block the translation of EBNA1; and mutation of Gly-Ala repeat domain abrogates the inhibition of EBNA1 translation.^163,164^ Hsp90 does not interact with EBNA1directly, a bridge protein may be involved in this process. Inhibiting EBNA1 expression strongly suppresses both EBV-induced primary B cell transformation in vitro and lymphoproliferative disease in SCID mice in vivo.^163,164^

#### Virus assembly

Only a few papers reported the function of Hsp90 in DNA virus assembly. The activated Hsp90 is needed for HBV assembly.^169,170^ Hsp90 enhances the affinity of core protein dimers for capsid formation and prevents capsid dissociation.^169^ Besides, the reactive oxygen species (ROS)-promoted HBV capsid assembly also requires an active form of Hsp90.^170^

#### Virus-induced tumorigenesis

As described before, EBV is the causative regent of several tumors, including Burkitt’s lymphoma^171^ and Nasopharyngeal carcinoma (NPC).^172^ The latent membrane protein 1 (LMP1) is regarded as an oncoprotein that promotes tumor metastasis and invasiveness through inducing the expression of matrix metalloproteinase 9 (MMP9), mimicking the tumor necrosis factor receptor (TNFR) superfamily proteins, and activating the NF-kB, MAPK, PI3K/Akt and JAK/STAT signal transduction pathways.^173,174^ Hsp90 seems positively promoting cell growth in EBV-positive nasopharyngeal carcinoma cells and EBV-infected T and NK cells.^175,176^ Hsp90 inhibitors, AT13387 and BIIB021, potently inhibit cell growth and induce apoptosis by impeding LMP1 function through activating its downstream signaling pathways described above.^175,176^

#### Immunity modulation

We have discussed how Hsp90 is hijacked by DNA viruses to promote viral replication above. Under certain conditions, Hsp90 exhibits antiviral activities by promoting cell immunity. In the acute infection stage, Hsp90 is induced to express in the cell surface of EBV-infected B cells. It has a strong ability to expand gamma delta T cells (γδ T cells) population.^177^ The γδ T cells have potent antiviral ability in the acute phage when a host is infected by HIV, influenza, Sendai, coxsackie, vaccinia, VSV, or HSV-1. The γδ T cells work as both early sentinels of the immune system by providing immediate protection and as bridging elements between innate immunity and adaptive immunity.^178^ Since Hsp90 can work as an immune sensor and assist antigen presentation, it may function in the same way in EBV infection.^177,179–182^

### The function of HSP90 family on cell transformation during retrovirus infection

Several HSPs functions as oncoproteins to promote cellular transformation. Hsp90 participates in the HTLV-1-induced cellular transformation. The *tax* protein of HTLV-1 controls viral replication and induces T lymphocyte transformation.^183^ NF-κB pathway is one of the main targets essential for this process;^184^ while Hsp90 acts as an important partner of *tax* by binding with and maintaining its stability in nucleus.^153,185^ Hsp90 inhibitors (e.g.,17-DMAG) or Hsp90-depletion by siRNAs cause *tax* degradation in proteasome, inhibition of NF-κB signalling, and activation of the long terminal repeat (LTR) of HTLV-1.^153,185^ Oral treatment with Hsp90 inhibitor 17-DMAG significantly suppresses the aggressive infiltration into multiple organs in ATL mice.^153,185^

## The function of HSP70 family in virus infection

### Functions of HSP70 family in RNA virus infection

#### Virus entry

Viruses in different families (e.g., Picornaviridae, Flaviviridae, and Reoviridae) take advantage of HSP70 family proteins for their entry into host cells. In the case of Picornaviridae, for example, the coxsackievirus A9 (CAV-9) uses Hsp70 homolog Grp78 for its entry.^186^ It shows that antibodies against Grp78 block 50% of virus binding. Integrin αvβ3 is another famous virus receptor.^187^ When cells are simultaneously treated with Grp78 and integrin αvβ3 antibodies, virus binding is blocked completely. Therefore, Grp78 functions as a co-receptor of CAV-9. Besides, Grp78 can interact with major histocompatibility complex (MHC) I molecules on the host cell membrane after infection of CAV-9. MHC I molecules help virus internalization into mammalian cells.^186^ In the course of EV-A71 infection, Hsp70 is dramatically upregulated and interacts with EV-A71 on the cell surface. Hsp70 antibody significantly inhibits virus binding to the cell surface.^188^ Besides enteroviruses, many viruses of Flaviviridae family also require Hsp70 to entry into host cells. By affinity chromatography assay, Hsp70 is discovered to form a complex with Hsp90 and DENV receptor that facilitates viral entry.^110,189^ Hsp70 interacts with DNEV envelope protein (E protein) and plays a significant role in virus attachment.^190^ Similarly, antibodies against Hsp70 and Hsp90 significantly inhibit DENV infection.^110,189^ The same mechanism is also observed in JEV infection.^191^ Hsp70 is enriched in the lipid raft and colocalized with the E protein in JEV-infected Huh7 cells.^192^ The depletion of cholesterol disrupts the enrichment and colocalization of the E protein and Hsp70 to a raft membrane. Eventually, it decreases JEV entry without any effects on virus attachment.^192^ These results suggest that Hsp70 works as a receptor of JEV; and lipid rafts serve as an organizing centre to facilitate JEV entry. At the late stage of JEV entry, Hsc70 (isoform D) is upregulated in C6/36 cells upon JEV infection. However, it seems that Hsc70 is not required for virus attachment to the cell membrane but needed for virus penetration into the host cells. It is suggested that Hsc70 holds an intense involvement in clathrin-mediated endocytosis at the late stage of viral entry, which helps JEV to penetrate into host cells.^193^ Recently, it is reported that Grp78 is also required for JEV both in the attachment and entry steps.^194^ Antibody targeting the N-terminal of Grp78 significantly prevents virus attaching to host cells whereas antibody targeting the C-terminus fails to block the attachment. Knockdown of Grp78 also inhibits JEV internalization. The colocalization and interaction between Grp78 and JEV envelope protein provide solid evidence to show the importance of Grp78 in the process of virus attachment and entry.^194^ Interestingly, Grp78 is secreted out of host cells after JEV infection, and the secreted Grp78 cooperates with JEV to promote virus infection.^195^ Recent studies show that Grp78 is a receptor of SARS-COV, MERS-CoV, and SARS-CoV-2 viruses.^196^ ZIKV infection is positively regulated by Hsp70 at multiple stages.^197^ Hsp70 inhibitors impair virus entry, RNA replication, and capsid assembly of different ZIKV strains in diverse cell lines.^190,197^ Rotavirus infection also needs the assistance of membrane-resident Hsc70.^198^ Hsc70-specific monoclonal antibodies inhibit virus internalization and infection without effect on virus attachment.^198^ Further evidence shows that the whole virus particle and a short domain (or a peptide) in the C-terminal region of VP5 is sufficient to bind to Hsc70.^199^ The ATPase domain of Hsc70 is proved to be necessary for its interaction with VP5 and induction of virion conformation change for the entry.^200^

#### Virus replication

HSP70 family proteins participate viral replication by employing different mechanisms. First, HSP70 family proteins facilitate the formation of virus replication complex and/or maintain the stability of complex proteins. In some cases, HSP70 family proteins directly interact with viral polymerase to enhance viral replication. For example, during the Mumps virus (MuV) infection, the expression level of Hsp72 is increased. The C-terminal region of Hsp72 interacts with the N-terminal region of P protein, which is an essential component of RdRp complex. Knockdown of Hsp72 results in accumulated ubiquitinated P protein as well as increased cell apoptosis.^201^ Besides, Hsp70 is also reported to regulate L protein, another MuV polymerase component. Hsp70 cooperates with Hsp90 to regulate L protein levels. Hsp90 inhibitor, 17-AAG, reduces the L protein level through promoting degradation via the C terminus of Hsp70-interacting protein (CHIP) -mediated proteasomal pathway. Hsp70 inhibitor VER155008 together with 17-AAG enhances L protein degradation. Therefore, Hsp90 and Hsp70 together regulate the stability of L protein and ensure the proper virus replication complex (VRC) formation.^202^ In the case of canine distemper virus (CDV) infection, the increased Hsp70 results in an elevated expression of light nucleocapsid (NC-L) variant, which displays polymerase activity.^203,204^ A more direct evidence is that Hsp70 facilitates viral RNA production in cell-free transcriptional assays.^204^ Furthermore, it is demonstrated that Hsp70 interacts with and regulates NC polymerase activity dependent on the Hsp70 ATP activity; because Hsp70 antibody significantly inhibits NC polymerase activity and supplementation of purified recombinant Hsp70 enhances both the basal and stress-induced NC polymerase activity.^205^

The other members of Hsp70s also regulate VRC formation. Hsp72 physically interacts with several replication proteins of Flavivirus including NS5A, NS3, and NS5B (RdRp). For example, Hsp72 participates the VRC formation of HCV.^206^ Downregulating Hsp72 leads to a decreased number of VRC in HCV-infected cells, while overexpression of Hsp72 raises the number of VRC.^206^ Hsc70 is associated with VRC by binding on the 3’ polyU/UC motif of HCV RNA genome.^207^ HCV accumulation and virion production are significantly suppressed when cells are treated with Hsp70 or Hsc70 inhibitors.^208^ Similar result is reported in the case of RSV infection. Ectopic expression of RSV nucleocapsid protein (N protein) and phosphoprotein (P protein) are detected to interact with Hsp70 in 293T cells.^209^ The N protein is responsible for interacting with the viral RNA, and P protein interacts with N protein and with the RdRp L to form the nucleocapsid. In RSV-infected cells, Hsp70 redistributes into lipid-raft membranes and colocalizes with virus N protein and lipid raft marker GM1.^210^ Although Hsp70 inhibitors suppress RSV polymerase activity;^210^ it only disrupts viral gene expression but do not affect RNA polymerization.^211^ Therefore, more detailed studies are needed to understand the functions of Hsp70 in modulating RSV gene expression and replication.

In term of Ebola virus (EBOV) replication, the mechanism of Hsp70 involved is much more complicated. By using immunoprecipitation and mass spectrometry assays, it has been identified that the N protein interacts with Hsp70, NEF, BAG2, and the Hsp70 co-chaperone DNAJA2.^212^ The N protein recognizes and binds to the viral RNA genome to establish a steady N protein–RNA complex structure (RNP). This complex further interacts with viral proteins VP30, VP35 and RdRp L, to finally form VRC. Here, Hsp70 functions to maintain the stability of N protein and helps to facilitate VRC formation.^212,213^ In addition, Hsp70 is also co-purified with L polymerase in insect cells.^214^ Besides, Hsc70 interacts with the terminal non-coding regions of the EBOV genome. Disruption of the interaction by mutating the binding site potently inhibits the minigenome replication of EBOV.^215^

Another mechanism that Hsp70 employs to support virus replication is to modulate nuclear import of polymerase or nuclear capsid. Some RNA viruses also replicate in the nuclear such as CDV and influenza virus. Therefore, nuclear transportation becomes a critical step for their replication. Upon CDV infection, Hsp70 is shown a strong contribution to viral replication by interacting with and promoting the translocation of the nucleocapsid particles from the cytosol to nucleus.^216^ Similarly, during influenza infection, Hsp70 interacts with PB2 or PB1 monomers and PB2/PB1 heterodimer in HeLa and HEK293T cells, and sequentially translocates into the nucleus with PB2 monomers or PB2/PB1 heterodimers.^217^ If Hsp70 and PB2/PB1 polymerases are retained in the cytosol, the polymerase activity reduces dramatically. The shuttling of Hsp70 between nuclear and cytoplasmic compartments underlies the modulatory effect of Hsp70 on influenza virus replication.^217^

#### Virus gene expression

Besides viral entry and replication, Hsp70 also contributes to viral protein translation. Positive single-stranded RNA viruses (e.g., SARS-CoV-2, HCV, ZIKA, EV-A71, etc) use the internal ribosome entry site (IRES) to initiate the translation of their own proteins but inhibit the host cellular cap-dependent translation through regulation or cleavage of eukaryotic translation initiation /elongation factors (EIFs/EEFs). In the case of coxsackievirus B3 (CVA B3) infection, Hsp70 is upregulated to enhance the initiation and elongation of viral translation.^218^ In the translation initiation step, Hsp70 upregulates IRES-acting factor lupus autoantigen protein expression and activates eIF4E binding protein 1 (EIF4EBP1), a cap-dependent translation suppressor. In the elongation step, Hsp70 activates the Akt-mammalian target of rapamycin complex 1 (mTORC1) signal cascade, leading to activation of EEF2 via kinase p70S6K- and Cdc2-mediated phosphorylation and inactivation of EEF2 kinase (EF2K).^218^ Hsc70 enhances the IRES activity in EV-A71 infected cells. Hsc70 interacts with 2A protease of EV-A71 to enhance EIF4G cleavage that impairs host cell cap-dependent translation but enhances viral IRES-mediated translation.^219^ Therefore, Hsc70 may serve as an antiviral target against EV-A71 and HCV infections. Some viruses do not have IRES sequence, and virus replication produces plenty of dsRNAs which trigger the activation of protein kinase-RNA-activated (PKR)- EIF2a signalling cascade that shuts down global translation in cells and releases stresses.^220,221^ To circumvent the PKR-mediated block to viral proliferation, influenza A virus induces the cellular tetratricopeptide repeat (TPR) - domains containing JDP protein, p58IPK. Influenza virus downregulates PKR in an Hsp70-dependent way.^222–226^ In uninfected and unstressed cells, p58IPK activity is clogged with Hdj1 by forming a complex.^225,226^ During influenza A infection, the amount of Hdj1 in p58IPK-Hdj1 complex decreases to an undetectable level. These findings suggest that the activation of p58IPK appears to be a sequel to the Hsp70-mediated release of Hdj1 from the p58IPK-Hdj1 complex, which allows the monomeric p58IPK to inhibit PKR.^225^ In the Hsp40 chaperone part, we would discuss more details about the regulation of PKR signaling by Influenza virus.

#### Virion assembly

Hsp70 is reported to assist some viruses’ assembly. During morphogenesis of the double-stranded RNA Reovirus, Hsp70 contributes to the assembly of trimeric sigma 1 protein, which is responsible for the interaction with host cell receptor.^227^ While the N-terminal segment of the sigma 1 protein folds and trimerizes cotranslationally in an Hsp70-independent manner, a post-translational fold of the C-terminal globular domain is dependent on Hsp70. In this process, Hsp70 binds cotranslationally to the region downstream the N-terminal α-helical coiled-coil, which presumably helps to inhibit unwanted interaction and misfolding. Trimerization of the C-terminal domain of the sigma 1 protein is coupled to the ATP-dependent release of Hsp70 from the ribosome.^227^ Besides, in HeLa cells infected by poliovirus or coxsackievirus B1, Hsp70 is detected to interact with the capsid precursor P1.^228^ In the complex, P1 is mainly newly synthesized and has a longer half-life than that of total P1. The Hsp70-P1 complex is regarded as an assembly intermediate of picornaviruses.^228^

Interestingly, some researchers demonstrate that Hsp70 inhibits influenza virus replication by blocking nuclear export of viral ribonucleoprotein complex (vRNP), and subsequent viral morphogenesis via disassociating M1 from vRNP.^229,230^

#### Virus release

The evidence of HSPs on virus releasing is limited. Both Hsp70 and Hsc70 can interact with NS5A protein; although they play different roles in HCV infection.^231^ Silencing of Hsp70 decreases viral protein expression, but the virus protein level is not affected.^232,233^ Instead, interfering Hsc70 reduces extracellular virion production.^232,233^ Moreover, Hsc70 is embedded in the viral capsid. And co-localization between Hsc70 and core and E2 structural proteins of HCV has been found in lipid droplets. Therefore, Hsp70 and Hsc70 may regulate HCV infection release at different steps.

### The function of HSP70 family proteins in DNA virus infection

#### Virus entry and genome releasing

Instead of virus attachment and entry into the host cells, here we talked about virus entry into the cytosol from ER. The cytosol entry from ER is a key step in SV40 infections. Hsc70 is reported to be essential in this step. Hsc70 interacts with and is regulated by SGTA.^234^ Further studies show that HSP70 superfamily member Hsp105 forms a subunit in the Hsc70-SGTA complex to facilitate SV40 cytosol entry.^235^ In addition, Grp78/Bip also plays a role in SV40 cytosol entry. Grp78 interacts with SV40 capsid protein in a DNAJB11-dependent manner to help SV40 disassemble and enter into the cytosol.^236^

Hsp70 is also needed for the genome release of some DNA viruses. Such a process is described in Adenovirus infection. After the release of the virion from endocytic vesicles into the cytoplasm, Hsp70 and Hsc70 immediately attach to the hexon protein, one of the major Adenovirus coat proteins.^237^ Hsp70/Hsc70 and its co-chaperone Bag3 interact with the penton base protein, the viral capsid constituent responsible for virus internalization.^238,239^ The intact nucleocapsid is transported to nucleus through the typical NLS-dependent nuclear import machinery.^240^ The nucleocapsid anchors to the nuclear pore through its hexon protein by interacting with components of the pore complex. Then viral DNA is transferred into the nucleus in a Hsp70-dependent manner but leaving hexon outside the nucleus because the purified hexon, instead of viral DNA, enter the nucleus in a Hsp70-independent manner.^240^ A possible explanation is that the intact nucleocapsid is too large to pass through the nuclear pore complex, while the disassembly of nucleocapsid facilitates the entry of viral DNA into nucleus. However, more solid evidence is needed for such an explanation.^238^ Other examples for the contribution of Hsc70 in viral genome release in host nucleus are HSV and Polyomavirus. In HSV-infected cells, the translocation of Hsc70 from the cytosol to nucleus is triggered by the immediate-early viral protein ICP0. Hsc70 is colocalized with the components of the 26S proteasome and virus UL6 portal protein, which provides the conduit for DNA entry and exit from the capsid. UL6 is highly ubiquitinated in the nucleus, indicating that Hsc70 may be responsible for the correct folding and degradation of UL6 in the ubiquitin-proteasome pathway, though there was no direct evidence that UL6 is a substrate of Hsc70.^241^ It is also believed that Hsc70 contributes to Polyomavirus genome nuclear import through its interaction with all viral capsid proteins VP1, VP2 and VP3 both in vitro and in vivo. Hsc70 translocates from the cytosol to the nucleus accompanied by the translocation of capsid proteins upon infection with Polyomavirus.^254^

#### Gene expression and protein maturation

Most viruses manipulate the cellular transcription and translation machinery and shut off host protein synthesis, so that they can take advantage of these machineries and recruit initiation and elongation factors for the expression of viral proteins. Some host factors exploited by viruses interact closely with components of Hsp70 complex. Therefore, the chaperone system is highly important for viral gene expression. Several transcription initiation factors are well known to physically interact with the Hsp70 co-chaperone Bag1 in vitro. Bag1 stimulates general transcription activity in an Hsp70-dependent manner.^242–244^ The stimulation of global transcription is detected in cells upon infections by either human polyomavirus, John Cunningham virus (JCV) or HCMV.^245,246^ However, the detailed molecular mechanism of the general transcriptional activation by viruses is to be further investigated.

The typical example of Hsp70 system in regulating the maturation of viral proteins is shown in HBV large envelope protein (LHBsAg).^247–249^ The mature LHBsAg has a unique dual transmembrane topology. Initially, the C-terminal of LHBsAg co-translationally resides in ER, while the N-terminus is resident in the cytosol and later is translocated into ER for post-tranreported that the expression of Grp78 is stimulatedslation. To ensure the correct topology, Hsp70 system strictly regulates the post translocation of N-terminal of LHBsAg. During the translation, Hsc70 interacts with the N-terminal of LHBsAg at amino acids 63 to 107 and suppresses LHBsAg translocation into ER.^248,249^ Hsc70 cochaperones Hip and Bag1 also regulate the activity of Hsc70 in an antagonistic way.^250,251^ Overexpressed Hip promotes Hsc70 activity resulting in more cytosol retention of LHBsAg N-terminal;^250^ while Bag1 overexpression could inhibit Hsc70 activity to promote nuclear translocation.^251^ In the post-translation process, Grp78 binds with LHBsAg and Hsc70 to facilitate the ER translocation of HBV large surface antigen.^247,248,252,253^ The function of Grp78 is regulated by both positive regulator ER-localized DnaJ-domain-containing protein 4 (ERdj4) and negative regulator BiP-associated protein (BAP).^253^ Increased BAP destabilizes LHBsAg/BiP complex.^253^ Together, Hsp70 chaperone system is crucial in modulating the sophisticated topogenesis of HBV envelope protein.^247^

#### Virus assembly

A lot of DNA viruses normally assemble in the nuclei of infected cells. Taking Polyomavirus as an example, all capsid proteins are synthesized in the cytosol, whereas subsequent assembly of virions only takes place in the nucleus. During Polyomavirus infection, the constitutive form of Hsp70 and Hsc70 are coimmunoprecipitated with all three viral capsid proteins (VP1, VP2, and VP3). Hsp70 is translocated from cytoplasm to the nucleus in the late stage of infection, coincident with localization change of the viral capsid proteins.^254^ In vitro studies show that Hsp70 functions to keep proper assembly of Polyomavirus.^255^ The prokaryotic Hsp70 chaperone DnaK also interacts with recombinant VP1 at the C-terminal domain where it links pentamers in an assembled capsid.^255^ When DnaK binds to VP1, it inhibits VP1 assembly, which is induced by calcium in vitro. However, combining the Hsp70 chaperone system including DnaK, DnaJ and GRpE with VP1 together is sufficient to assemble VP1 into uniform capsids in the presence of ATP alone without calcium.^255^

### The function of HSP70 family in retrovirus infection

Human T lymphotropic virus type 1 (HTLV-1) is a well-investigated example of retrovirus that interacts with Hsp70 proteins. During HTLV-1 infection, syncytium formation is a key factor for cell-to-cell virus transfer. The syncytium formation is subject to close cell-to-cell interactions.^256,257^ Cell membrane-resident Hsc70 is necessary in this process as Hsc70-specific monoclonal antibodies eliminates the syncytium formation and HTLV-1 infectivity. The same outcome presents when cell is treated with a peptide derived from the HTLV-1 glycoprotein gp46, which binds to Hsc70.^258,259^ Interestingly, although Hsc70 enhances the syncytium formation, it has no effect on virus entry.^259^

Hsp70 also plays an important role in the post-entry steps. During human immunodeficiency virus type 1 (HIV-1) infection, viral protein R (Vpr) stimulates interaction between the viral preintegration complex and karyopherin-alpha to facilitate viral nuclear import. Hsp70 functionally overlaps with Vpr in this process.^260^ When Vpr is deficient, Hsp70 could rescue virus nuclear import by interacting with karyopherin-alpha at the N-terminal that also binds Vpr. Interestingly, some researchers argue for the antiviral role of Hsp70 because Hsp70 and Vpr share the same substrate. It seems that Hsp70 would compete and inhibit Vpr function. Since HIV-1 needs Vpr to manipulate cell cycle and apoptosis, Hsp70 neutralizes the function of Vpr in HIV-1 infection.^261,262^ Recently, HIV is observed to package Hsp70 as part of virion core. The virion-incorporated Hsp70 ATPase activity and correct conformation of Hsp70-virion are essential for HIV infection, since inhibition of Hsp70 ATPase activity interrupts the Hsp70-virion core association and diminishes virus infectivity.^263^

### The effects of HSP70s on host cells upon DNA virus infection

#### Cellular transformation

**Fig. 5:**
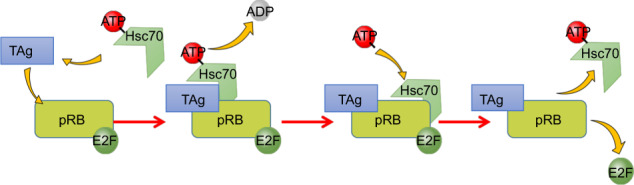
Model for the participation of Hsc70 in the TAg induced disassembling of pRBand E2F. Firstly, TAg combines with pRB-E2F complex to facilitate T-antigen-Hsc70 complex formation. Then TAg-Hsc70 complex stimulates ATP hydrolysis, and Hsc70 transfers to the high-affinity conformation allowing the trap of the substrate protein pRB or E2F. Finally, Hsc70 induces the conformation of the substrate protein in the complex which assists the dissociation of pRB and E2F to initiate cell cycle reprogram

DNA viruses those do not encode polymerase in their genome are dependent on host DNA replication machinery. To replicate in quiescent cells, the virus has to reinitiate cell cycle thereby transforming the host cells. Some mechanisms have evolved in enabling viruses to overcome the restriction points of cell cycle. The best-investigated example is SV40. The large and small T antigen (TAg) are central for the SV40 transformation ability. At their N terminus, both types of TAg contain the J-domain, the signature motif of an Hsp70 co-chaperone. Mutation or deletion of the J-domain disrupts the functional association of TAg with Hsp70s. The ability of TAg to transform mammalian cells is subsequently obliterated.^264^ Also, TAg sequesters the retinoblastoma family proteins (pRb, p107, and p130) and liberates members of the E2F family of transcription factors in a Hsc70-ATP hydrolysis-dependent manner.^265,266^ The free E2F family proteins subsequently trigger the expression of the S-phase genes leading to DNA replication.^267^ The above observations are interpreted in the following four steps. Firstly, the large TAg combines with pRB-E2F complex. Subsequently, the pRB-E2F-TAg complex associates with Hsc70 in the presence of ATP as Hsc70 ATP-bound form presents high substrate association rate to facilitate TAg-Hsc70 complex formation. The third step is that the J-domain of TAg in the TAg-Hsc70 complex stimulates ATP hydrolysis. This process is dependent on the active site of Hsc70 and does not occur at the T-antigen binding site. Meanwhile, Hsc70 transfers to the high-affinity conformation allowing the trap of the substrate protein pRB or E2F. In the last step, Hsc70 induces the conformation of the substrate protein in the complex which assists the dissociation of pRB and E2F. After ADP dissociation and rebinding of ATP to Hsc70, E2F and the pRB-TAg complex is released from the Hsc70–substrate complex.^268–271^ A second strategy of TAg to transactivate E2F transcription is independent of the disruption of the pRB-E2F complex that also involves the J-domain and Hsp70 protein.^271–274^ TAg functions like Bag1 to assiste the assembly of a transcription initiation complex on the respective promoters in the presence of Hsc70. Alternatively, TAg could induce Hsc70 to disassemble an inhibitory silencer complex or to assist with remodelling the chromatin (Fig. 5).

HPV and Adenovirus have similar transforming activities by disrupting pRB-E2F complexes. Although neither E7 (HPV) nor E1A (adenovirus) protein contains a J-domain, both proteins could transform cells in a way similar to that described for SV40 TAg. E7 interacts with tumor suppressor hTid-1.^275^ The C terminus of E7, which mediates the interaction with hTid-1, is essential for the physical disruption of the pRB-E2F complex though it is not necessary for direct interaction with pRB.^276,277^ These observations suggest that the interaction with hTid-1 is involved in the disruption of the pRB-E2F complex, providing E7 with the J-domain necessary to recruit Hsc70 for the complex dissociation in analogy to the function of SV40 TAg. Alternatively, the binding of E7 to hTid-1 could transform cells through inhibition of the assumed tumor suppressor function of hTid-1. E1A directly interacts with Hsc70 to disrupt the pRB-E2F complex.^278^

In conclusion, most double-stranded DNA viruses depend on Hsp70 chaperones for the reprogramming of the host cells to re-enter the cell cycle.

#### Cell survival and apoptosis

Since Hsp70 systems are essential modulators for cell survival under stress conditions, the induction of Hsp70 protein facilitates virus infection by keeping the cell alive until mature viruses are ready to leave. This is the main reason why the disruption of apoptotic pathway is a quite common phenomenon in viral infections.^279–281^ In the early stage of viral life cycle, viral reproduction is simply vulnerable to cell death. Naturally, viruses are evolved in manipulating cellular apoptosis. In EBV-infected cells, nuclear oncoprotein EBNA3A helps Hsp70 nuclear translocation and Hsp70 chaperone complex formation to immortalize B cells through inhibiting apoptosis.^282^ In contrast, apoptosis is beneficial for virus spreading when virions are finally assembled.^283–290^ Virions are found in apoptotic bodies and subsequently engulfed by phagocytic cells. It has been suggested that virus can infect neighbour cells without being detected by the host immune system.^291,292^ On the other hand, the decrease of Hsp70 mRNA level may lead to the timed induction of apoptosis at the late stage of adenovirus infections.

#### Innate immunity

Hsp70 has been previously described to influence innate immunity and inflammatory responses.^293^ It has been believed that hepatocyte is devoid of innate immunities. Ma et al. reported that the expression of Grp78 is stimulated by HBV replication. The elevated Grp78 protein in turn activated innate immune response by induction of IFNβ expression.^294^ Further studies showed that Hsp70 greatly contributes to cellular innate immunity in response to either virus or bacterial infections. In the course of bacterial infections, the elevated intracellular levels of Hsp70 protect cells from LPS-mediated inflammation. Through its interaction with TNF Receptor Associated Factor 6 (TRAF6), Hsp70 can inhibit its ubiquitination and thereby block the activation of transcription factor NF-kB.^295,296^ Weiss et al. has presented more details on this mechanism. Hsp70 binds with IKK, leading to disturbing the function and stability of NF-κB/IkBα/IKK complex and further impairing IkBα phosphorylation.^297^ The disturbance of this complex also affects IkBα proteasomal degradation and nuclear translocation of NF-κB complex.^298^ The inhibition of NF-κB signalling has great therapeutic significance because it can prevent massive tissue damage medicated by the excessive inflammation response.

A more recent study provides another approach for Hsp70 to regulate inflammation. Pierre et al. described an NF-κB independent pathway that Hsp70 could impact NLRP3/ASC inflammasome formation through its association with NLRP3.^299^ Aside from the anti-inflammation function of intracellular Hsp70, the secreted extracellular Hsp70 binds to dendritic cells and macrophages before being recognized by its binding elements, most of which are innate immune receptors.^293,300^

Toll-like receptors (TLRs) detect virus invasion and immediately trigger intracellular innate antiviral response.^301^ They belong to type I integral membrane glycoproteins of IL-1 receptor (IL-1R) superfamily.^302^ It has been suggested that TLR2/TLR4 involved in the initiation of innate immunity by extracellular Hsp70. Hsp70 utilizes both TLR2 and TLR4 to transduce its proinflammatory signal in a CD14-dependent manner to promote proinflammatory cytokine production via MyD88/IRAK/NF-κB axis signalling cascade.^8,301^ Another study clearly demonstrated the function of TLR4 and its direct interaction with Hsp70.^303^

After ligand binding, TLRs dimerize and undergo a conformational change required for recruiting downstream signaling molecules, including the adaptor molecule myeloid differentiation primary-response protein 88 (MyD88), IL-1R-associated kinases (IRAKs), TGFβ-activated kinase (TAK1), TAK1-binding protein 1 (TAB1), TAB2 and TNF-receptor-associated factor 6 (TRAF6).^304–307^ Many other innate immunity-related signaling pathways would then be activated, for example, phosphorylation of NF-κB via TAK1/IKK activation. MAPKs p38, JNK, and ERK pathway are also activated, then subsequently activate CREB and AP-1 transcription factors. Both AP-1 and NF-κB activate proinflammatory cytokine expression, including TNFα, IL-6, IL-1β, and a number of other cytokines and chemokines.^308–311^

## The functions of HSP60 family in virus infection

### The effects of HSP60s on host cells upon RNA virus infection

#### Immunity modulation

Few studies show the function of Hsp60s in the life cycle of RNA virus; however, the function of Hsp60 in regulating host immunity has been widely studied.^312–316^ The majority of studies show that Hsp60 works as an activator of immune response. In the case of JEV infection, Hsp60 facilitates virus-induced inflammation by promoting IL-1β production via increasing NLRP3 inflammasome activity and NFκB phosphorylation.^317^ However, under certain conditions, viruses also utilize Hsp60 to evade host cell immune response. The genome-wide RNA interference (RNAi) screen identifies the interaction of Hsp60 with PB2 of influenza virus.^318^ Hsp60 helps PB2 translocation from the cytosol into mitochondria.^38,39^ Then the mitochondrial PB2 interacts with and modulates the activity of mitochondrial antiviral signaling protein (MAVS) to suppress interferon β (IFNβ) production.^38^ Therefore, Hsp60 determines the effect of PB2 on both mitochondrial stability and the level of IFN-β production.^38,39^ Besides, DENV infection also elevates Hsp60 expression. Silencing of Hsp60 results in an increase of IFN-α production and decrease of virus reproduction in macrophages.^319^ However, the detailed mechanism remains elusive.

#### Apoptosis regulation

Viruses use different mechanisms to modulate apoptosis. In the case of HCV infection, ROS production is regarded as the major contributor of HCC although it also promotes cell apoptosis.^320^ Study shows that the N-terminal domain of HCV core protein can induce ROS production by interacting with Hsp60 and inhibiting the normal function of Hsp60 in releasing protein stress.^321,322^ While another RNA virus, Rotavirus SA11, tries its best to delay the early apoptosis through modulating Hsp60 stability.^323^ Hsp60 helps to translocate NSP4 protein of Rotavirus into mitochondria from cytosol and induces apoptosis.^323^ To yield more viruses, Rotavirus infection increases the Hsp60 phosphorylation at Tyr^228^ by activated Src kinase that leads to the ubiquitin-mediated proteasomal degradation.^323^ Even though virus postpones apoptosis via Hsp60, the main function of Hsp60 is to refold proteins in mitochondria.^323,324^

### The function of HSP60 family in DNA virus infection

#### Virus replication

In the previous sections, we have discussed that Hsp90 is important for RT-ε RNA complex formation in HBV infection. However, it has been shown that Hsp60 directly regulates RT activity before the RT-ε RNA complex formation.^325,326^ HBV replication is markedly suppressed when Hsp60 is knocked down by specific siRNAs.^325^ Hsp60 transiently interacts with RT to activate RT in an ATP-dependent manner.^326^ Upon RT activation, Hsp60 immediately dissociates from the Hsp60/RT complex without being encapsidated into viral nucleocapsid.^326^ More detailed research shows that at least one of two RT fragments, residues 1–199 of terminal protein (TP) domain and 680–842 of Rnase H (RH), is necessary for Hsp60 binding.^327^ The TP domain is also responsible for the binding of Hsp90 in the RT-ε RNA priming step.^138^

#### Apoptosis regulation

Similar to HCV infection, HBV infection also induces strong apoptosis which is thought mainly contributed by HBx protein.^328^ Studies show that overexpression of Hsp60 facilitates HBx-induced apoptosis.^329,330^ The interaction of HBx and Hsp60 has been observed and confirmed by different methods including affinity purification, mass spectrometry and co-immunoprecipitation.^329^ Hsp60 binds on a small domain (residues 88–117) of HBx.^329^ Furthermore, Hsp60 also forms a complex with HBx and Hsp70 in the mitochondria.^330^ However, the mechanism of how Hsp60 enhances the apoptosis remains unclear during HBV infections.

#### Immunity modulation

Hsp60 is involved in both the innate and adaptive immune response.^314^ Here, we focus on how HBV harnesses Hsp60 to evade host immune response. Numerous studies show that HBV employs active means to escape innate immune response and induce immunosuppression.^331^ Among these strategies, HBV infection increases the population of CD4^+^CD25^+^ T regulatory cells (Tregs) which can produce an amount of IL-10 and TGF-β.^332^ IL-10 is also called cytokine synthesis inhibitory factor (CSIF) displaying anti-inflammatory properties.^333^ It influences both the first and the second line of immune defence.^334^ HBV infection increases serum sHsp60 level and makes use of Hsp60 to activate CD4^+^CD25^+^ regulatory T via TLR2/MyD88/IL10 signaling.^335^

### The function of HSP60 family in retrovirus infection

Although the role of Hsp60 in DNA/RNA virus infection process has been widely studied; only limited knowledge has been obtained in retrovirus infection. Hsp60 is encapsidated into HIV particles,^336^ but we have no idea about its function. The integrase catalyzes the integration of HIV-1 pro-viral DNA in the host genome.^337^ A small portion of Hsp60 colocalizes and interacts with the viral integrase (IN).^338^ Hsp60-Hsp10 complex maintains the integrase at an active form and stimulates its activity in an ATP-dependent manner.^338^ The integration is a critical step for successful infection of HIV-1.

## The function of HSP40 (Hsp70 co-chaperones) in virus infection

### The function of HSP40 family in RNA virus infection

#### Virus replication

Hsp40s regulate RNA viral replication by modulating polymerase activity, replication complex and nuclear transportation. In JEV-infected cells, Hsp40/DnaJ homolog Hdj2 interacts and colocalizes with NS5 protein, an RdRp essential for viral RNA genome replication.^339^ Overexpressed Hdj2 promotes JEV replication significantly. However, how Hdj2 modulates NS5 activity remains elusive.^339^ Influenza virus also takes advantage of Hsp40 to promote replication by assisting VRC to relocate into nucleus. The replication of influenza virus occurs in the nucleus of host cells; therefore, nuclear trafficking of viral ribonucleoprotein (vRNP) complex is required. The vRNP is composed of viral RNA (vRNA), polymerase heterotrimer (PA, PB1, PB2) and nucleoprotein (NP).^340^ NP has a key function of interacting with importins through its nuclear localization signals.^341,342^ Hsp40s have two strategies to help vRNP transport into nucleus. First, Hsp40/DnaJB1 interacts with NP at the early stage of infection and ensures efficient association between NP and importin alpha.^343^ This interaction is mediated by the J domain of Hsp40 and the N-terminal region of NP.^343^ Another strategy is that Hsp40/DnaJA1 binds with the PB2 and PA polymerase subunits, then co-translocates into nucleus with PB1-PA complex.^344^ Besides, Hsp40/DnaJA1 enhances viral RNA synthesis both in vivo and in vitro.^344^ Different from DnaJB1, DnaJA1 mainly depends on its C-terminal substrate-binding domain instead of typical J domain to manage viral RNA synthesis.^344^ In the replication process, Hsp70 is reported to enhance polymerase nuclear translocation.^217^ Thus it is proposed that DnaJA1 cooperates with Hsp70 and assist RNA polymerase nuclear import.^217,343^

#### Virus gene expression

Viral RNA replication produces plenty of double-stranded RNA (dsRNA) molecules. Host cell detects these dsRNA and activates interferon-induced protein kinase (PKR) to restrict viral replication by phosphorylating eukaryotic initiation factor eIF2α and preventing protein synthesis.^220,221,345^ However, in order to escape from the antiviral response of host cells, the influenza virus smartly blocks the activation of PKR/eIF-2α. Influenza virus NS1 directly binds the N-terminal of PKR and inhibits PKR activation.^225^ Besides, NP protein also interacts with Hsp40, leading to dissociation of Hsp40 and p58IPK.^346^ It is reported that type III Hsp40/p58IPK is an inhibitor of PKR,^222–224,226,347^ while Hsp40 is the inhibitor of p58IPK.^225,348^ Therefore, the dissociation of Hsp40 results in activation of p58IPK. Subsequently, p58IPK inhibits the activity of PKR/eIF-2α. As a result, influenza virus releases the inhibition of protein synthesis. However, in the late stage of infection, influenza virus M2, Hsp40 and p58IPK form a stable complex that would lead to PKR activation, ER-stress-induced cell death and virion release.^349^

#### Protein maturation

Flavivirus genome encodes a large polyprotein which is later cleaved into several mature structures and non-structure proteins. The mature proteins then form VRCs. At the beginning, HSP40 family protein DNAJC14 participates in the VRC formation of flavivirus. During yellow fever virus (YFV) infection, DNAJC14 is recruited to non-structural protein clustering sites with NS3 and NS5 to form VRC.^350^ However, either knockdown or overexpression of DNAJC14 inhibits YFV and HCV replication.^350,351^ Later, it has been demonstrated that DNAJC14 overexpression affects YFV polyprotein processing and alters VRC assembly. Overexpression of DNAJC14 alters the cleavage sites of NS3/4A and NS4A/2K and gives rise to abnormal NS3 to NS3-4A ratios, suggesting that the chaperone activity of DNAJC14 modulates NS3/4A/2K cleavage that ensures appropriate expression level of NS3 and NS4A. The inhibition of VRC formation upon ectopic expressing DNAJC14 is caused by chaperone dysregulation.^351,352^

#### Immunity modulation

Hsp40s sometimes act to help the virus evade host immunity, while sometimes it exhibits antiviral activity by increasing host immune response under certain conditions. It is reported that the expression of DNAJB1/Hsp40 and Hsp70 is induced by PolyI:C stimulation.^353^ Hsp40 cooperates with Hsp70 to suppress the MDA5/MAVS pathway though interacting with MDA5 and inhibiting MDA5 multimer formation.^353^ However, DNAJA3 shows its ability to suppress virus replication. During HFDV infection, VP1 is able to suppress the type I interferon signaling via suppression of phosphorylation, dimerization, and nuclear translocation of IRF3.^354^ However, DNAJA3 induces lysosomal degradation of VP1 protein. Therefore, DNAJA3 indirectly stimulates the immune response of host cells.^354^

### The function of HSP40 family in DNA virus infection

#### Virus replication

Evidence suggests that Hsp40s regulate the initiation of DNA virus replication. In the case of HPV replication, it starts with the recognition of protein E2 on the origin (*Ori*) sequence and recruitments of replication initiator E1, which displays ATPase and helicase activities.^355–357^ Hsp40 (Hdj1 and Hdj2) and Hsp70 enhance E1 binding on the *Ori* independently and additively. Hsp40 directly binds with E1 and remains in the E1-ori complex, whereas Hsp70 transiently interacts with E1 in an ATP-dependent manner.^355^ Subsequent study reveals an additional role of Hdj2 in facilitating E1 helicase function by replacing E2 in the E1/E2/*Ori* complex.^358^ The stable association of E2 to *Ori* flanking the E1 binding site may act as a DNA clamp to prevent DNA unwinding. Similarly, Hsp40 (hTid1) also has similar functions to Hdj1 and Hdj2 with independent chaperone activity. Hsp40 interacts with HSV-1 replication initiator protein and helicase protein UL9, thereby promotes their binding to the replication origin.^359,360^ This provides another example of the involvement of J-proteins in the replication process of eukaryotic DNA viruses.

Except for enhancing HBV replication, a possible negative role of Hsp40 has also been reported. The core protein is a key component of viral capsid and essential for virion assembly, while HBx is a multifunctional virulence factor implicated in viral replication and hepatocarcinogenesis in human. A yeast 2-hybrid approach is used to identify interactions between the core protein and two Hsp40s, Hdj1 and hTid1.^361,362^ Individual expression of each Hsp40 in hepatocytes transfected with a replication-competent HBV construct shows an inhibitory effect on both viral replication and capsid formation. Further studies reveal that both core and HBx proteins are destabilized by co-expression with Hdj1 or hTid1; because they are targeted for enhanced proteolytic degradation. Except inhibiting HBV replication, Hdj1 also facilitates proteasome-mediated degradation of HBx.^361^

#### Virus induced cellular transformation

As mentioned above, some viruses can induce cell transformation and tumorigenesis. Here, we discuss the role of Hsp40 proteins on virus-induced cellular transformation. Hsp40 may have a negative role in HBV-induced cell transformation. It has been demonstrated that HBx is the major factor for induction of hepatocyte transformation.^363–366^ Nevertheless, overexpression of Hsp40s (Hdj1 and hTid1) significantly enhance the proteasome-mediated degradation of HBx. Another study suggests that hTid1 interacts with E7 oncoprotein and promotes the cellular transformation by HPV16;^275^ because the binding sequence of E7 shares high similarity to the TAg oncoproteins of SV40.

### The function of HSP40 family in retrovirus infection

#### Nuclear entry of viral pre-integration complex

HIV can efficiently infect nondividing cells.^367^ This requires active transport of the viral pre-integration complex (PIC) into the nucleus without breaking down nuclear membrane. Some components of PIC implicated in regulating nuclear import include the central DNA flap and viral proteins IN, MA, and Vpr of HIV-1 (or Vpx of HIV-2).^288,368–372^ Hsp40/DnaJB6 interacts and enhances the nuclear localization of Vpx as well as promotes the nuclear import of viral PIC.^373^ Similarly, DnaJB6 also promotes Vpr nuclear localization during HIV-1 infection;^374,375^ particularly, the long isoform of DnaJB6 is extremely important in this process.^375^ The expression level of DNAJB6 S/L isoform is regulated by the polyadenylation factor CstF64. High level of CstF64 favors DNAJB6-S isoform production, whereas a low level of CstF64 enhances DNAJB6-L isoform production.^374^

#### Regulation of viral gene expression

A notable example of Hsp40 involvement in regulating retrovirus gene expression is its importance in enhancing the gene expression during HIV-1 infection.^376^ Nef protein, an important viral protein associated with pathogenesis and disease progression, stimulates Hsp40 expression by enhancing Hsp40 promoter activity via HSF1 transcription factor.^377–379^ Hsp40 and Nef co-translocate into nucleus where they become a part of CDK9-associated transcription complex to enhance long terminal repeat (LTR) mediated gene expression.^376,380^ The binding of HSF1 on HIV-1 LTR promoter induces viral gene expression directly.^380^ Interestingly, Hsp70 seems to act contrary to Hsp40, which also presents in the Nef-Hsp40 complex. Different from Hsp40, Hsp70 suppresses viral replication and gene expression; while Hsp40 rescues the Hsp70-downreguled viral gene expression. Hsp70 inhibits CDK9 phosphorylation, an essential event for high-affinity binding of HIV-1 transactivator of transcription-positive transcription elongation factor b complex for transactivating response RNA.^381^ It is also reported that some other Hsp40 family proteins negatively regulate HIV replication. Hsp40A1, B1, B6 and C5 (but not C3) are able to limit HIV-1 production, while they have no effect on viral gene expression upon infection by adenovirus, HSV-1 or vaccinia virus. The conserved DNAJ domain is suggested to be responsible for the inhibiting HIV-1 reproduction. The Hsp70/Hsp40 complex specifically recognizes and inhibits the Rev translation or accelerates its degradation, leading to inhibiting viral gene expression.^382^

## The function of sHSPs in virus infection

Small heat shock proteins are the most upregulated proteins identified in host cells under stress conditions, for example, when cells are exposed to elevated ROS level, abnormally high temperature, or pathogen invasions.^383^ In most cases, sHSPs are responsible for recognizing misfolded proteins and transferring them to other ATP-dependent chaperones for proper folding, or proteasomes or autophagosomes for degradation.^49,384^ Hsp27 is one of the most ubiquitously expressed sHSPs with the highest level in skeletal, smooth, and cardiac muscles.^59,385^ Like all other sHSPs, Hsp27 shares a highly conserved α-crystallin domain, or so-called C-terminal domain. It contains 6-8 β-strands, forming 2 β-sheets as intermolecular interaction sites.^41,59^ Because of the importance of Hsp27, in this section, we mainly focus on the function of Hsp27 in virus infection.

Hsp27 has been shown particular importance in viral infections. Rajaiya et al. suggested that the association of Hsp27 with p38 or NFκB/p65 plays key roles in controlling the expression of pro-inflammatory mediators in virus-infected cells.^64^ Fukagawa et al. suggested that the phosphorylated Hsp27 is upregulated through the PI3K/Akt pathway upon EBV infection.^386^ We would discuss the special roles of Hsp27 in different viral infections in the following sections.

### The function of Hsp27 in RNA virus infection

**Fig. 6:**
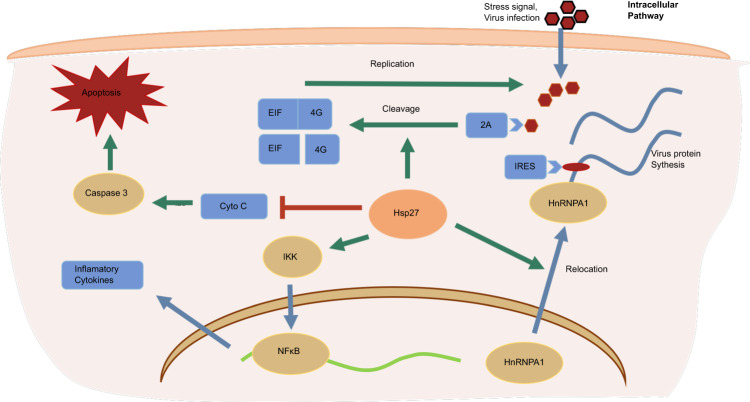
The controversial function of Hsp27 during virus infection. Hsp27 would enhance 2A protein-mediated eIF4G cleavage, which would in turn promote IRES function and viral genome replication. It correlates with hnRNPA1 translocation from host cell nuclei to cytosol, and the consequential virus protein translation. Hsp27 is also able to suppress apoptosis via caspase 3 inhibition. On the other hand, Hsp27 could help activating IKK complex, modulating NF-κB and AP-1 to induce the expression of pro-inflammatory cytokines

Hsp27 is upregulated during EV-A71 infection by proteomics analysis. Knockout of Hsp27 results in the suppression of virus replication and protein expression level, while their restoration appears after Hsp27 is restored. Also, Hsp27 enhances viral IRES activity by promoting 2A^pro^ - mediated EIF4G cleavage.^57^ Interestingly, the nuclear translocation of Hsp27 from cytosol is reversely correlated with the relocalization of RNA chaperone hnRNPA1 from nucleus to the cytosol for initiating viral protein translation upon EV-A71 infection. However, knockout of Hsp27 blocks hnRNPA1 cytosol relocolization, indicating a fundamental role of Hsp27 in regulating the import/export of nuclear proteins during virus infection (Fig. 6).^383^ Hsp27 is rapid upregulated at the early stage (4 hours post infection) of coronavirus infection,^387^ suggesting an important role in virus early replication and possibly a good target for treating SARS-CoV-2 infection. Swine fever virus (CSFV) is a member of the family Flaviviridae. Hsp27 is found to bind with NS5A, which is a non-structural protein in response to viral replication and assembly. Hsp27 depletion enhances the virus replication while the replication is suppressed by ectopic expression of Hsp27 through activating NF-κB signaling in PK-15 cells.^388^ Porcine epidemic diarrhoea virus (PEDV) infection causes high fatality in swine. The Hsp27 is obviously upregulated in PEDV infected MARC-145 cells.^389^ However, the virus titer is declined by ectopic expression of Hsp27. Hsp27 could activate NF-κB signalling and enhance the transcription of IFNβ and downstream interferon stimulated genes (ISGs).^389^

### The function of Hsp27 in DNA virus infection

Pro-inflammatory cytokines play crucial roles in early antiviral infections. Adenovirus infection causes a wide variety of diseases.^390^ The internalization of Adenovirus leads to the activation of ERK1/2, which could in turn trans-activate NF-κB and AP-1 to induce pro-inflammatory cytokine IL-8 expression in different experimental systems.^391–393^ A study shows that downregulation of Hsp27 leads to an increased release of IL-8 from keratinocytes stimulated with UV or TNFα. The increase of IL-8 is suppressed by NF-κB inhibitor and correlated with an enhanced IκB-α degradation and phosphorylated IκB-α accumulation in Hsp27-depleted cells. In addition, Hsp27 is shown to be associated with the IκB kinase (IKK) complex. Because the synthesis of prostanoid PGE2 and IL-8 is regulated by NF-κB, it could be a likely mechanism of Hsp27 in modulating inflammatory cytokine production.^70^ Further studies also show that Hsp27 is of particular importance in the cyclooxygenase-2 and IL-6 expression via activating p38 MAPK/MK2 signalling and the consequential stabilization of cyclooxygenase-2 and IL-6 mRNAs.^68^

Aside from its involvement in pro-inflammatory response regulation, researches have also found the antioxidant role of Hsp27 in regulating stress caused by ROS.^49^ It is commonly agreed that Hsp27 regulates enzyme activities by upholding glutathione in reduced form, such as glutathione reductase and glucose-6-phosphate dehydrogenase.^394^ More recent evidence correlates the level of sHSPs with the intracellular content of iron, a catalyzer of hydroxyl radical generation. Hsp27 also exhibits an important role in oxidized protein degradation machinery.^395,396^ However, there is also controversial evidence indicating the involvement of Hsp27 in accumulation of oxidized proteins that benefits herpesvirus replication. Experimental models are set up using two distantly related herpesviruses Rhesus Rhadinovirus (RRV) and HSV-1. They are close relative of Kaposi’s sarcoma-associated herpesvirus (KSHV). The oxidized proteins are accumulated during these viral infections. Results show the removal of only a part of oxidized proteins in a proteasome-dependent manner, while some others resisting degradation.^397^ Oxidized proteins resisting proteolysis become sequestered in foci within the nucleus and coincided with Hsp27-enriched foci; although they are not associated with virus-induced chaperone enriched domains (VICE). Furthermore, the accumulation of oxidized proteins is more pronounced in Hsp27-depleted cells.^398^ One possible explanation is that Hsp27 buffers the toxic effects of those defective proteins undergoing proteolysis through aggregation in the nucleus. The roles of Hsp27 are most likely not mutually exclusive during virus infection.

Hsp27 also contributes to virus replication. Porcine circovirus type 2 (PCV2) is a single-stranded DNA virus that causes the postweaning multisystemic wasting syndrome (PMWS) in pigs. The phosphorylated Hsp27 is upregulated in the nucleus in PCV2-infected PK-15 cells. Hsp27 inhibitors such as SB203580 suppress PCV2 replication. The same result appears upon Hsp27 knockdown. In contrast, ectopic expression of Hsp27 promotes viral replication.^399^ Moreover, the phosphorylation of Hsp27 is also upregulated in EBV-positive cells, as well as the phosphorylated (activated) Akt levels. When EBV-positive cells are treated with PI3K inhibitors, the phosphorylated Hsp27 level is decreased, suggesting that the phosphorylation of Hsp27 is upregulated through the PI3K/Akt signalling pathway upon EBV infection.^386^ However, studies also show that Hsp27 can both positively and negatively regulate the virus replication depending on the virus/cell types. Tong et al. reported that Hsp27 works as an antiviral protein against HBV replication through enhancing IFN production in hepatocytes. The Hsp27 expression level is increased in both HBV-infected human liver tissues and HBV-producing HepG2.2.15 cells.^400^

## The function of PDIs in virus infection

### The function of PDIs on virus entry and uncoating

The entry of some viruses into eukaryotic cells is governed by redox-regulated processes. One example is newcastle disease virus (NDV), a bird virus in the family of paramyxoviruses. This negative-sense, single-stranded RNA paramyxovirus gains entry to its host cell through large conformational changes in its fusogenic F-protein, which involves thiol/disulfide exchange.^401^ Overexpression of PDI and ERdJ5 (a PDI family reductase with an extra J domain) leads to an increase of viral membrane fusion, indicating a route whereby virus can take advantage of the PDI family to gain access to host cells.^402^ In endothelial cells surface, PDI possibly reduces β1 and β3 integrins allowing DENV entry.^403,404^ Also, thiol blockers and PDI inhibitors decrease the entry of rotavirus in MA104 cells, indicating the involvement of thiols for infectivity.^405^

The entry of HIV is regulated by PDIs. The envelope of HIV becomes unhinged by PDI for entry. Ryser et al. firstly reported that cleavage of two disulfide bonds in the gp120 surface component of the HIV-1 envelope is required for virion entry into CD4^+^ cells. In this process, the PDI on cell surface is responsible for this effect.^406^ PDI inhibitors sufficiently prevent the reduction and block the cleavage of surface-bound disulfide conjugate, thereby prevent infection at the level of HIV-1 entry.^407^ Now, there are numerous studies on how envelope binds on host cells. Results from different groups have demonstrated that gp120 moves laterally along the membrane surface until it collides with a patch of PDI in a domain of the membrane that distinguishes from a typical lipid raft. PDI reduces two disulfide bonds in gp120, producing conformation changes that likely stabilize the binding of gp120 to CD4 and expose the V3 loop for subsequent binding to the chemokine coreceptor. Following this, gp41 undergoes rearrangement into its fusogenic intermediates and entry occurs.^408,409^ During the entry, Galectin-9 binds PDI to regulate the redox environment on the cell surface and enhance HIV entry.^410^ Taken together, the reports from these three groups have profound implications for our understanding of the HIV virion surface structure and viral entry.

The other examples are the nonenveloped Polyomavirus (Py). Py particles contain a layer of coat protein VP1. This single protein, arranged as 72 pentamers, forms the shell surrounding the viral genome.^411^ After internalization, Py penetrates the ER membrane to gain access to the cytosol and then the nucleus for viral genome transcription and replication. PDIs isomerize or reduce the virus disulfide bonds to generate a membrane transport-competent intermediate for host cell membrane penetration.^412^ For example, SV40 entry involves caveolar/lipid raft-mediated endocytosis, vesicular transport to ER and translocation into the cytosol. ERp57 isomerizes the interchain disulfides connecting VP1 for virus uncoating.^413^ The further study demonstrates that ERp57 and PDI operate in concert with ERp29 to unfold the VP1 C-terminal arm. PDI and ERp72 reduce Py, while ERp57 principally isomerizes the virus in vitro. Mutagenesis study subsequently identified that the residues C^11^ and C^15^ of VP1 are important for infection, suggesting a role for these residues during isomerization.^414^

### PDIs regulate viral protein translation

A little evidence shows that PDIs are involved in viral protein translation. Some positive single-stranded RNA (+ssRNA) virus depends on IRES-mediated translation for viral protein synthesis. EV-A71 infection is potently inhibited by an active compound Oblongifolin M (OM), which is isolated from herb *Garcinia oblongifolia*. Further studies show that OM suppresses the viral IRES-mediated translation of polypeptide via suppressing ERp57, and ectopic expression of ERp57 increases the IRES activity and partially rescues the decreased viral replication caused by OM treatment.^415^ The detailed mechanism how ERp57 downregulates IRES activity would be further investigated.

### PDIs regulate viral activities by influencing oxidative stress and ER stress

Several studies have demonstrated the implication of redox balance disruption in the establishment of viral infection and the progression of virus-induced diseases. And accumulated ROS in turn may modulate the viral replication and cellular response that also contribute to viral pathogenesis.^416,417^ Virus-induced oxidative stress has been reported during HIV,^418^ influenza virus,^419^ HBV,^420^ HCV,^421^ encephalomyocarditis virus (EMCV),^422^ respiratory syncytial virus (RSV),^423^ and JEV^424^ infections.

Considering its thioredoxin-like sites, ERp57 has been thought a main player in the mechanisms of cell protection against oxidative stress, regardless of its subcellular location. Redox proteomics analysis of HPV positive tissues shows that the expression level of ERp57 and GST is positively correlated with tissue redox status, suggesting its potential association with viral-induced oxidative DNA and protein damages.^425^

**Fig. 7:**
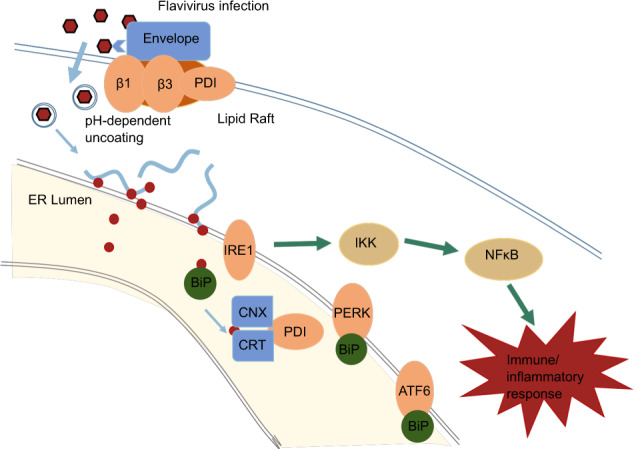
An example of PDI function and ER stress response during Flavivirus infection. Flavivirus entry into endothelial cells could be suppressed by silencing of PDI. Studies also show that PDI co-localizes with cell surface lipid rafts along with flavivirus envelope protein, leading to the activation of cell surface integrins (b1 and b3), which has direct implications in aiding the virus entering host cells. Viral RNA is then released and located around ER for translation. The increased protein synthesis may disturb ER homeostasis, leading to unfolded protein response through GRp78/BiP dissociation with PERK and ATF6 to activate PERK, ATF6, and IRE1. Downstream responses include activation of NF-κB and other signal pathways, followed by immune and inflammatory responses

ER stress is another consequence caused by virus activities. Viral infection would lead to exploitation of the ER membrane, accumulation of misfolded proteins, and imbalance of calcium concentration. Influenza A virus (IAV), HBV, JEV, DENV, and ZIKA virus all hijack host cell process to enhance viral pathogenesis, such as facilitating viral folding and trafficking, affecting receptor interaction, and modulating host immune responses.^426–428^ Therefore, as a major factor in ER stress response, ERp57 would be critical for viral protein glycosylation and maturation, which may in turn affect virus release and infection (Fig. 7).

## The function of RNA chaperones in virus infection

### The Functions of RNA chaperones in RNA virus infection

The life cycle of most RNA viruses is completed in the cytoplasm of host cells. To complete the life cycle, virus is often able to induce redistribution of many host cell proteins; particularly, those proteins involved in RNA metabolism and functional regulation of viral RNAs. hnRNPs, such as hnRNP A1,^429^ hnRNP C1/C2,^430^ hnRNP D^431^ and hnRNP I,^432^ are mainly resident in the nuclear but quite often shuttle to cytoplasm for stabilizing the viral RNA and initiating cap-independent translation.

Viruses employ various means to redistribute hnRNPs. For instance, the EBOV inhibits the nuclear import of hnRNP C1/C2. VP24 binds to NPI-1 subfamily karyopherin-alpha nuclear import proteins at the C-terminal region (amino acids 424–457) and prevents their interaction with tyrosine-phosphorylated STAT1 (pSTAT1) and hnRNP C1/C2. The inhibition results in cytoplasm retention of pSTAT1 and hnRNP C1/C2.^430^ Alternatively, virus may redistribute hnRNPs by promoting nuclear export. Rae1 is an mRNA export factor. VSV infection induces hnRNP A1 cytoplasm redistribution in a Rae1-dependent manner; and the redistributed hnRNPA1 is responsible for VSV-induced apoptosis.^431^ While the cytoplasm translocation of AUF1 is dependent on 2A protease (2A^pro^) and virus RNA replication.^433,434^ Cytoplasmic translocation of hnRNPA2 is induced by core protein. HnRNP A2 binds to negative-stranded RNA of JEV and facilitates virus replication.^435^ However, the detailed mechanism of how 2A^pro^ and core protein induce the redistribution remains unknown.

#### Virus replication

It has been well documented that hnRNPs greatly contribute to the virus replication. For example, hnRNP I/PTB and hnRNP C participate replication of different viruses, e.g., coronavirus,^436^ HCV^437^ and poliovirus.^438^

The role of hnRNP I/PTBs in viral RNA synthesis seems to vary among different viruses. PTBs bind UCUAA pentanucleotide repeats at IRES and 3′-UTR. PTBs modulate coronavirus RNA synthesis.^436^ However, the function of hnRNP I/PTB is to some degree complicated in HCV RNA replication. It selectively interacts with the 3′ end of the HCV genomic RNA. The upstream SL2 and SL3 stem-loop structures are essential for hnRNP-I/PTB I binding whereas the most 3′-terminal stem-loop SL1 is not needed. HnRNP I/PTB I and hnRNP C specifically bind the pyrimidine-rich region of in the 3′NTR of HCV RNA genome.^437^ Possibly, hnRNP I/PTB I is recruited for helping to initiate viral negative-strand (−) RNA genome replication, to stabilize RNA genome, and/or to regulate the encapsidation of genomic RNA.^439,440^ Up to date, the detailed function of hnRNP I/PTB I binding on 3′UTR has not been elucidated. Some studies showed that PTBs have an inhibitory effect on the synthesis of HCV RNA genome,^441^ while Aizaki et al. reported that PTBs are required for efficient HCV RNA replication.^442^ It has been postulated that they suppress the initiation of viral genome RNA replication but enhance IRES-mediated translation or facilitate the replication–translation switch. It is shown that HuR can compete hnRNP I/PTB I binding on 3′ UTR of the viral RNA to facilitate La binding to the 3′ UTR; while La protein is critical for HCV genome replication.^443–445^

Although hnRNP C has many similarities with hnRNP I and both bind with the 3′UTR of the HCV RNA genome to facilitate viral RNA replication;^437^ more details demonstrate that only hnRNP C binds the 3′-ends of viral RNAs with both negative and positive polarities.^446^ Besides, hnRNP C also binds at the 5′- end on negative-strand RNA of poliovirus to facilitate the synthesis of positive-strand RNA;^438^ while miR-555 decreases the expression of hnRNP C thereby inhibits poliovirus replication.^447,448^

#### Virus RNA splicing

The main function of hnRNPs is modulating RNA process and metabolism, including splicing, stabilization and transportation. It has been documented that hnRNP K helps the RNA splicing of IAV. IAV is a major human pathogen with a genome comprised of eight negative-stranded RNA segments. Two viral RNA segments, NS1 and M, undergo alternative splicing and yield several proteins including NS1, NS2, M1, and M2 proteins. Two of the influenza virus RNA segments generate spliced products: NS segment codes for non-structural protein NS1 and nuclear export protein NEP/NS2; M segment encodes the matrix protein (M1) and ion channel (M2). NS1-BP properly splices the viral M1 mRNA segment to yield the M2 mRNA without affecting the splicing of M4 mRNA or NS mRNA segments. In this process, hnRNP K works as a mediator to bridge the interaction of NS1-BP (binding protein) and M mRNA. Lack of neither NS1-BP nor hnRNP K ensures the proper splicing of M mRNA.^449,450^ Further studies show that NS1-BP and hnRNP K bind M mRNA downstream of the M2 5′ splice site (5′ss). NS1-BP binds most proximal to the 5′ss, partially overlapping the U1 snRNP binding site, while hnRNP K binds further downstream and promotes U1 snRNP recruitment. Mutation of either or both the hnRNP K and NS1-BP-binding sites results in M segment mis-splicing and attenuated IAV replication.^451^

#### Virus translation

Virus RNA translation often harnesses host cellular proteins. Most hnRNPs positively regulate virus translation. Positive-sense single-stranded RNA viruses normally contain an IRES sequence that drives viral protein translation independence of the cellular cap-dependent translation. hnRNPs bind with the IRES of different viruses to assist their translation. During HCV virus infection, hnRNP I, hnRNP L, hnRNP D,^452–454^ hnRNP A1 and hnRNP K^455^ all participate the translation of HCV viral protein by binding with 5′UTR sequence. hnRNP I (PTB3), is one of the polypyrimidine tract-binding proteins (PTBs) that has been reported to bind viral RNA recurrently. They could bind the IRES of picornaviruses, including cardio-aphthovirus group (including EMCV, EV-A71) and entero-rhinovirus group (poliovirus and HRV-2).^89,456^ PTBs also bind HCV UTR regions,^441^ calicivirus RNA^457^ and coronavirus RNAs.^436^ For picornavirus and HCV, it is proposed that PTBs could help IRES folding into a translation-competent structure.^458^ Although unlike canonical transient interactions shared by RNA chaperones, it is not clear whether PTBs are eliminated after the IRES has folded properly.^459^ PTB binds the 5’ UTR of HCV RNA genome to initiate translation. In translation assays, PTB antibody efficiently blocks the IRES-mediated translation in vitro.^460^ Three RRM motifs of PTB monomer directly bind with IRES of FMDV to stabilize IRES structure and enhance eIF4G entry to IRES.^459^ hnRNP L is capable of binding the 3′ border of the IRES.^461–463^ hnRNP L binds with single stranded-HCV RNA when preannealing single-stranded RNA with miR-122.^463^ HnRNP D, also referred to as AU-rich element RNA-binding protein 1 (AUF1), shuttles between the cytosol and nucleus. It is found that hnRNP D could also interact with the stem-loop II of HCV 5′ UTR, and its overexpression enhances HCV IRES-dependent translation.^452^ PIM1 inhibitors induce hnRNP D relocalization from nucleus to cytosol so that it binds the IRES and inhibit EV-A71 protein translation.^122^ Similarly, hnRNP K interacts with SL1 located in the 5′ -UTR of HCV genome where is bound with miR-122 binding site.^464,465^ miR-122 is required for HCV replication. It binds at a conserved sequence in the 5′ -UTR and increases the stability of HCV RNA.^466–468^ It is also reported that residues 25–91, a hydrophilic region near the N terminus of HCV core protein, binds to proline-rich domains of hnRNP K and negatively regulates the effect on human thymidine kinase transcription.^469^ However, its function on viral replication is not well addressed.

While hnRNP A1 binds with both 5′- and 3′-UTR of HCV RNA, they form a complex with NS5b and septin 6 to assist viral protein translation. The C-terminal of hnRNP A1 and N-terminal of septin-6 are required in the translation process. Since hnRNP A1 has many homologous such as hnRNP A/B, hnRNP A2/B1, and hnRNP A2; all of them may substitute for hnRNP A1 in regulating IRES-mediated translation.^470^ The enteroviruses’ IRES sequence also interacts with hnRNPs. hnRNP A1 specifically binds on the 5′-UTR of EV-A71 and enhances IRES-dependent translation.^471^ Apigenin, a dietary flavonoid, interacts with hnRNPs and interferes with their RNA editing activity.^472^ The binding of hnRNP A1 with viral RNA is significantly blocked when the cells are infected with EV-A71 upon treating with Apigenin, leading to marked suppression of IRES-mediated translation. It is noted that hnRNP A1 redistribution is not affected in this experiment. Recent studies show that hnRNP A1 cytoplasmic translocation is strictly regulated by some signalling or stress proteins, such as MINK/p38 MAPK pathway^473^ and Hsp27.^57^ P38 inhibitors or Hsp27 knockout dramatically block hnRNP A1 translocating from nucleus into cytosol, leading to inhibition of virus replication.^57,473^

Except for promoting viral protein translation by binding with the viral IRES sequence, hnRNPs can directly bind with virus proteins to facilitate virus replication. Upon CVB3 infection, 3C^pro^ binds and cleaves hnRNP M to facilitate virus replication.^474^ However, hnRNP M does not affect IRES activity and RNA stability.^475^ hnRNP K is required for DENV replication.^476^ The core protein of DNEV interacts with hnRNP K to release the inhibitory effects of hnRNP K on transcriptional activator C/EBPb.^477,478^ Other studies demonstrate that hnRNP C1/C2 interact with viral RNA and NS1 protein of DNEV and facilitate virus reproduction.^479–482^ hnRNP D binds on both 5′- and 3′-UTR of enteroviruses and inhibits translation without affecting RNA decay.^434^ On the other hand, virus also applies strategy to release the inhibitory effects of hnRNP D via protease 3CD cleavage.^434^ The cytoplasm translocation of hnRNP D is dependent on the expression of 2A^pro^ and viral RNA replication.^433,434^ hnRNP D also inhibits nucleocapsid expression by interacting with an AU-rich sequence (nt 41 to 60) in the 3′ UTR of NIV.^483^

#### Virus RNA export

hnRNP A2/B1 interacts with NS1 of influenza virus, leading to decrease of the viral NS1 RNA/protein levels as well as NS1 RNA nuclear export.^484^

#### RNA polyadenylation

The *gag* gene of Rous sarcoma virus contains a cis-acting negative regulator of splicing (NRS) element. The general function of NRS is usually manifested by binding serine/arginine-rich (SR) protein hnRNP H and U1/U11 snRNPs, resulting in inhibition of splicing by acting as a decoy 5′ splice site. Evidenced by in vitro polyadenylation analysis, a new function of NRS element is revealed that it is required for 3′ LTR polyadenylation. In this process, hnRNP H binds on NRS element and promotes polyadenylation; however, mutation of the binding sites of U1 and U11 snRNPs to the NRS does not affect polyadenylation.^485,486^ An opposite result shows that polyadenylation stimulatory activity of NRS is dependent of U1 and/or U11 snRNP as well as SR proteins; while hnRNP H seems not participating in the splicing control or Rous sarcoma virus polyadenylation.^487^

### The functions of RNA chaperones in regulating viral activities of DNA viruses

#### Virus replication

The transient lytic DNA replication of HCMV relies on the cis-acting element oriLyt, six viral-encoded core proteins, the proposed DNA replication initiator protein UL84, IE2, IRS1 and the gene products from the UL112/113 loci. Here it is reported that hnRNP K is sufficient to interact with UL84 protein of HCMV thereby promotes viral replication. The interaction is further enhanced by another two virus proteins UL44 and IE2.^488^

#### Gene expression

hnRNPs regulate DNA virus gene expression at both transcriptional and post-transcription levels either positively or negatively.

At the transcription level, hnRNP D0B and hnRNP A/B are capable of binding with cis-acting AAGTATGCA core element of HPV18 late promoter to suppress the late genes L1 and L2, which are components of virus capsid proteins.^425,489^ hnRNP K binds enhancer II (ENII) to promote HBV replication. Ectopic expression of hnRNP K augments HBV replication; while hnRNP K knockdown significantly decreases HBV viral load.^490^ Further study reveals that APOBEC3B forms a super complex with hnRNP K that alters the ENII binding activity via conformational change, therefore suppresses the S gene promoter activity.^491^ In the course of HSV infection, the immediate early protein IE63 (ICP27), hnRNP K and casein kinase 2 (CK2) together form a big complex. CK2 is capable of phosphorylating hnRNP K and ICP27. The phosphorylated ICP27 is responsible for its nucleocytoplasmic translocation and interaction with hnRNP K. Up to date, the function of the complex formation is not well elucidated.^492,493^ It may affect ICP27 to recruit the cellular RNA polymerase II for the transcription of certain late genes.^494–496^

At the post-transcription level, hnRNPs regulate the polyadenylation, splicing, and translation during DNA virus infections. HPV genome can be divided into an early region and a late region, followed by the proximal early (pAE) and the distal late (pAL) polyadenylation signals, respectively.^497,498^ The virion production mainly depends on the differentiation-dependent induction of L1 and L2 late genes. It has been shown that hnRNP H downregulates the late gene L2 at an early stage by interacting with the multiple GGG motifs located 174 nucleotides downstream of the early polyadenylation signal pAE. The hnRNP H binding promotes polyadenylation at early polyadenylation signal and inhibits the L2 mRNA production, since L2 mRNA production need read-through into the late region and polyadenylation of the late transcripts at the pAL.^499^ While at the late infection stage, hnRNP H is captured by L1 to release the inhibitory effect on L2.^499,500^ This process is called late gene autoregulation which enables rapid viral capsid protein production.^500^ Another example for hnRNPs modulating the polyadenylation is that SM polymerase (pol) mRNA of EBV early protein is cleaved and polyadenylated inefficiently.^501^ Under certain conditions, EBV early protein SM may harness hnRNP A1 and hnRNP C to help the processing of polymerase mRNA.

Viral RNA splicing is also modulated by hnRNPs. hnRNP A1 negatively regulates the expression of HPV late genes by affecting RNA splicing. It directly binds with the late regulatory element (LRE) in differentiated HPV16-infected cells.^502^ hnRNP A1 binds with splicing silencer element to suppress the use of the 3′ splice site located immediately upstream of AUG at Late 1 (L1) mRNA. hnRNP A1 inhibits the splicing of late mRNAs through the splicing silencer sequence and prevents the premature expression of L1 gene.^503–505^ On the other hand, there is another 17 nucleotides resident immediately downstream of the splice site which counteracts the effect of hnRNP A1-binding splicing silencers.^506^ hnRNP I also interferes with the splicing inhibitory elements locating at the upstream and downstream of major late 5′ splice site SD3632, thereby activates the late gene expressions.^507^

At the translation level, hnRNP I and hnRNP K inhibit the translation of HPV late gene L2 via binding on a specific cis-acting element in the 3′ end of L2 mRNA;^508^ whereas the inhibition of L2 translation can be disrupted by c-Src-mediated phosphorylation of hnRNP K at multiple tyrosine residues.^509^

#### Cellular transformation

hnRNPs also exhibit some other functions during virus infections. AUF1 works as a major component of C promoter binding factor 2 (CBF2) to interact with EBNA2 and mediate EBNA2 targeting to the latency C promoter (Cp) of EBV, thereby inducing B-cell immortalization and viral latency in humans.^510^ AUF1 also binds with the EBER1 noncoding RNA of EBV. In EBV-positive cells, EBER1 is abundant; therefore it may compete with AUF1-interacting targets in the host cells.^511^ Both EBNA2 and EBER1 are proposed to facilitate cell transformation.

#### Immunity modulation

Aside from affecting virus life cycle directly, hnRNPs are able to regulate viral activities through modulating immune response. During HSV-1 infection, hnRNP A/B form a complex with viral DNA, followed by homodimerization and demethylation. These events result in translocating the complex to cytosol and activating natural immunity through type I interferon signalling. Moreover, the complex promotes N6-methyladenosine modification and cGAS-STING-related mRNAs translocation upon infection by DNA viruses, further enhancing the immune response for virus elimination.^512^

### The function of RNA chaperones in regulating the viral activities of retroviruses

As we have mentioned above, hnRNP A1 can bind to RNA/proteins and participate intracellular nucleo-cytoplasmic transportation,^513^ as well as alternative splicing of mRNA in eukaryotes. During retrovirus infection, hnRNPs participate in viral RNA transcription,^514^ splicing,^515–517^ translocation^516^ and translation.

#### Transcription

In HIV-infected cells, viral *nef* protein is required for high-level viral replication.^518^ It is reported that Nef, Eed, kinase Lck and nPKC subfamily (PKCδ/θ) form a complex NAKC (Nef-associated kinase complex) responsible for promoting viral replication.^519^ Later on, it is found that hnRNP K is also a partner of the complex. It bridges the interaction of Nef, Eed and the kinases. The hnRNP K-nucleated complex activates ERK1/2 and results in suppressing HIV promoter, enabling suboptimal amounts of Tat and transcription factors (e.g., NF-kB) for initiation of transcription.^514^

#### Post-transcription

HIV-1 takes advantage of alternative splicing to generate doses of messenger RNAs to encode the various viral proteins. It is known that over 40 message RNAs are created by alternative splicing from a single pre-mRNA.^520,521^ Alteration of splicing patterns dramatically affects the infectivity and pathogenesis of HIV‐1.^521,522^

The splicing of HIV mRNA is mainly controlled at the early stages of spliceosome assembly on pre-mRNA by a stepwise association of the small nuclear ribonucleoprotein particles (snRNPs) U1, U2, and U5·U4/U6. The early steps include the recognition of the 5′ splice site and the branch point sequence by U1 and U2 snRNPs, respectively.^523^ RS splicing factors, which contain a domain rich in arginines and serines (RS domain), assist these steps. U2AF, one of the splicing factors which consists of two subunits U2AF65 and U2AF35, interacts with the polypyrimidine tract and the 3′ splice site, respectively.^524^ Then the interaction mediates the association of U2 snRNP with the branch point sequence.^525–527^ SR splicing factors are essential for virtually every step of spliceosome assembly, including early recognition of splice sites, recruitment of basic splicing factors to the pre-mRNA, and formation of bridging contacts with other RS domain-containing splicing factors.^528^

Prior to forming spliceosomes, the pre-mRNA is packed with hnRNPs.^529^ It has been documented that pre-mRNAs bind with different subsets of hnRNPs, indicating sequence specificity at some degrees. A direct role of hnRNPs in constitutive splicing has not been observed; although it seems that the binding of hnRNPs exhibits an unspecific nature. It is widely believed that hnRNPs employ crucial functions of modelling pre-mRNA structure and initiating recognition of splice sites.^530^

The cis-acting sequence elements of cellular and viral pre-mRNAs undergoing alternative splicing regulate the process either positively or negatively. They bind trans-splicing factor machinery together and form splicing complex. The majority of trans-acting factors are either hnRNPs or SR family proteins. These proteins also regulate alternative RNA splicing either positively or negatively. SR proteins often regulate splicing in a positive way while some hnRNPs mainly do the job in a negative way.^528,530^

Alternative incomplete splicing of HIV-1 genomic mRNA produces more than 40 unique viral mRNA species within an HIV-1-infected cell through highly accurate regulation by *cis* exon splicing silence elements (ESS) and *trans* hnRNPs. The production of Tat, Rev and Vpr proteins is highly controlled because they play key roles in HIV-1 multiplication. ESS2 is the first identified ESS in the HIV-1 genome that locates downstream of 3′ ss A3 within exon 4 and specifically represses *tat* mRNA splicing.^531^ ESS2 is mapped to a 10 nt core sequence CUAGACUAGA.^532^ ESS3, which locates at Exon 7, represses splicing at 3′ ss A7.^533^ Fine structure mutagenesis indicated that ESS3 is bipartite; and each sub-elements [AGAUCC (ESS3a) and UUAG (ESS3b)] inhibits splicing independently.^534^ A third ESS (AUAGUUAGUCCUAGG, ESSV), which locates downstream of 3’ ss A2 in exon 3 within the *vif* coding sequence, represses splicing at 3′ ss A2.^535^

To modulate the expression of Tat protein, several hnRNPs participate the splicing process by interacting with these ESS, intron splicing silencer (ISS) elements directly or interfering enhancer splicing element (ESE) activity. For example, hnRNP A1 and hnRNP K synergistically bind on ESS2 and inhibit the utilization of A3 splicing site.^536,537^ The UAG triplets in ESS2 is required for hnRNP A1 binding, and several hnRNP A1-binding sites are also found at SLS3. The C-terminal Gly domain of hnRNP A1 is important for the binding. SR proteins SC35 and SRp40, the strong activators of site A3, have similar binding sites to hnRNP A1. Hence, they may compete with each other to bind ESS2 and ESE2.^536^ Another study shows that hnRNP H displays an inhibitory effect on splicing. hnRNP H binds to ESS2 and competes with U2AF35 for binding to the exon sequence flanking 3′-splice site A3. This binding results in the inhibition of splicing at the 3′-splice site A3.^538^ hnRNP A1 also inhibit *tat* splicing by binding with ISS, which is independent of exon splicing silencer (ESS2) and blocks the entry of U2 snRNP but not U2AF65.^539–541^ The UP1 domain of hnRNP A1 is responsible for the ISS binding, while the RGG domain of hnRNP A1 is not needed for the alternative splicing activity.^542,543^ In addition to ESS2 and ISS elements that are regulated by hnRNP A1, blocking the binding of SR protein SC35 on ESE is another strategy for the virus to inhibit the Tat expression at the early infection stage. hnRNP A/B proteins bind the ESE locating at *tat* exon 2 to repress splicing, whereas SC35 binds the ESE to activate splicing. The binding sites of hnRNP A1 and SC35 are overlapping within the juxtaposed ESE/ESS9. It seems that hnRNP A1 inhibits the splicing of the upstream intron by binding to the ESS and directly masking the SC35 binding site.^544,545^ Similarly, hnRNP A1 is also reported to compete with ASF/SF2 at ESE3/(GAA)3. Therefore, the ratio of ASF/SF2 to hnRNP A1 determines the utilization of ESE3/(GAA)3 for activation or repression at site A7.^515,541,546^

The expression of other HIV proteins is also regulated by hnRNPs via modulating splicing. hnRNP A/B proteins inhibit the splicing at 3′ splice site A2 which is used to generate *vpr* mRNA. hnRNP A/B proteins bind with ESSV with a consensus sequence PyUAG. The splicing at splice site A2 is increased when the three PyUAG motifs within ESSV are mutated, leading to increased *vpr* mRNA levels and reduced skipping of the noncoding exon flanked by A2 and D3.^547^ Others reported that hnRNP D also binds at ESSV and functions as an inhibitor of splicing.^548^ The binding of hnRNP A/B proteins at ESSV blocks U2AF65 binding to the PPT of the repressed 3′ splice site and inhibits the splicing efficiency of 3′ splice site.^549^

Interestingly, hnRNP H is found to positively regulate the exon 6D splicing of HIV mRNA. It interacts with the sequence CGGA and enables U1 snRNP assembly onto exon 6D.^550,551^ Further study shows that hnRNP H family proteins function as a splicing enhancer through enhancing the ATP-dependent spliceosomal complex formation.^552^

#### RNA translocation

After transcription and splicing, lots of spliced and unspliced RNA molecules of HIV exist in the nucleus. Translocation of RNA into the cytoplasm is dependent on the Rev protein. On one hand, some hnRNPs facilitate RNA translocation. In HIV-1 genome, two sequences similar to the hnRNP A2 response element (A2RE) function as cis-acting RNA trafficking sequence that binds to the trans-acting trafficking factor hnRNP A2. The binding mediates a specific RNA trafficking pathway characterized extensively in oligodendrocytes. A2RE-1 locates within the major homology region of *gag* gene; while A2RE-2 locates at a region overlapped between *vpr* and *tat* coding sequence. hnRNP A2 binds to both A2REs in vitro to form a complex, which is necessary for RNA transportation in oligodendrocytes in vivo. A2RE-mediated RNA transport requires both microtubule and hnRNP A2. If *gag* and *vpr* RNAs containing A2RE-1 and A2RE-2 respectively are differentially labelled, it is observed that they are co-assembled into the same RNA trafficking granules and cotransported to the periphery of the cell. Although the *tat* RNA contains A2RE-2, it is not transported as efficiently as *vpr* RNA.^553–555^ hnRNP D also has a similar function in assisting RNA translocation.^556^ On the other hand, hnRNP A2B1, hnRNP C and hnRNP U can retain the HIV-1 genomic RNA in nucleus. Depletion of hnRNP A2B1 results in cytoplasmic redistribution of the virus RNA genome in the absence of *rev.*^557,558^ An N-terminal fragment of the hnRNP U specifically targets the 3′ long terminal repeat (3′LTR) and blocks the cytoplasmic accumulation of mRNAs, thereby affecting HIV gene expression.^559^

Besides HIV, the regulatory protein ORF57 of Kaposi’s sarcoma-associated herpesvirus (KSHV) also interacts with hnRNP K. CK2 can phosphorylate ORF57 and promote ORF57-hnRNP K interaction. The ORF57-hnRNPK-CK2 complex may be important for RNA export of KSHV since ORF57 is responsible for the nuclear export of viral mRNAs.^560,561^ hnRNP A1 interferes with the binding of Rex to rex-response element (XRE). The Rex protein of HTLV-1 mediates the nuclear export of unspliced and incompletely spliced viral mRNAs. This process partly depends on the binding of Rex to rex-regulatory sequences XRE.^562^

#### Viral protein translation

HnRNP A1 is induced to redistribute into the cytoplasm in the late infection stage of HIV and enhances the IRES-mediated translation.^563^ hnRNP D helps the redistribution of HIV mRNA into the cytoplasm, and p45 and p42 isoforms increase viral Gag protein synthesis while p40 and p37 suppressed this process.^556^ hnRNP I interacts with a novel IRES within a latently expressed gene (vCyclin) of KSHV and enhances vFLIP expression.^564^

## Challenges of targeting stress proteins for antiviral therapy

### HSPs, ER stress and human diseases caused by virus infections

The network of HSPs and their co-chaperones is essential for cells to maintain protein homeostasis, including nascent protein folding, protein translocation across membranes, and protein complex formation. Many stress stimuli can disrupt protein homeostasis such as thermal stress, nutrient starvation, chemical toxicity, oxidative stress, hypoxia, inflammation, and virus infection.^565–568^ Stresses can lead to protein misfolding and aggregation that need to be resolved quickly to prevent cell and tissue damage.^568^ HSPs and their partners may facilitate protein degradation when cells cannot cope with massive damaged proteins.

Many lines of evidence suggest HSPs as major factors for protein surveillance to protect host cells against virus infections. It was observed that Hsp70/Hsp90 expression increased dramatically in HSV-infected cells.^569^ Overexpression of Hsp70 inhibits the translocation of viral capsid into nucleoli during flavivirus infections. Although the interaction has not yet been investigated in natural infection, the *in vitro* findings have suggested that Hsp70 may act as a negative regulator of viral capsid protein to protect host cells against WNV infection by abolishing cytotoxic effects induced by the viral capsid.^570^ Studies from ours and other groups show that almost all HSP subfamily members (Hsp90s, Hsc70, Hsp70, Grp78, Erp57, Hsp27) are highly responsible to enterovirus infection and play key roles in all stages of virus life cycle. Not surprisingly, demonstrated that all most all HSPs (Hsp90s, Hsp70s, Hsp60s, Hsp40s and Hsp27) participate coronavirus infection,^145,196,387,571^ suggesting good target for anti-COVID-19 drug development.

As discussed in above sections, HSPs exhibit immuno-modulatory effects on both innate and adaptive immune responses.^572–574^ HSPs are capable of regulating not only intracellular innate immunity, but extracellular innate and adaptive immunity as well. HSPs released from tumor cells can bind to surface receptors on antigen presenting cells (APCs) and elicit tumor-specific killers by means of antigen cross-presentation.^575,576^ For example, Hsp27 positively modulates NF-κB phosphorylation, increases IFNβ transcription and downstream antiviral interferon-induced genes (ISGs). PEDV infection downregulates Hsp27 expression to escape host antiviral surveillance.^389^

Additionally, extracellular HSPs can also regulate cytokine production by dendritic cells (DCs), underscoring a connection between innate and adaptive immune responses modulated by HSPs.^577^ The purified or recombinant Hsp70 can stimulate the production of pro‐inflammatory cytokines and C–C chemokines in monocytes, macrophages and DCs, and upregulate MHC and costimulatory molecules in DCs.^578^ Binding of extracellular Hsp72 to human monocytes and dendritic cells can induce the production of the pro-inflammatory cytokines including TNF-α, IL-1β, IL-6 and IL-12 and IFN-γ.^579^ In the process of above HSP-induced cytokine production, HSPs act as internal stimulus of the CD14/TLR (TLR2 and TLR4) complex signal transduction pathways that further activates NF‐κB and MAPKs signalling.^580,581^

Although the main functions of HSPs are to protect cells upon stresses, they are often hijacked by many viruses to achieve successful infections. Recent study has demonstrated that DENV NS3 protein acts as a viral suppressor of RNA silencing by interacting with Hsc70, thereby interrupting host antiviral system by RNAi pathway and subsequently enhancing DENV replication.^582^ EV-A71 takes advantage of HSPs (Hsp90, HSc70, ERp57, and Hsp27) to enhance 2A^pro^-mediated eIF4G cleavage that is favour viral protein translation and blocks host protein translation.^57,219,415^

Viruses also initiate ER stress in host cells after infection. They have to manipulate UPR activation leading to cell survival, rather than inflammation induction, autophagy and apoptosis. DENV modulates UPR activation in a sequential manner to prolong the viral life cycle by allowing cellular adaptation to cope with the infection-induced ER stress. UPR is transiently induced by PERK pathway resulting in phosphorylation of eIF2α and subsequently translational attenuation in the early phase of infection. This transient event allows viral protein synthesis and accumulation that finally trigger UPR by IRE1-XBP1 (X-box binding protein 1) axis in the mid-phase of infection. This results in the increased expression of Grp78 to facilitate protein folding and also the increased expression of GADD34 (growth arrest and DNA damage 34), which dephosphorylates eIF2ɑ, and thus allowing protein translation to be continued. The increased Grp78 also prevents cellular apoptosis-mediated by CHOP (pro-apoptotic protein during stress persistence). Finally, the increased viral proteins transiently trigger ATF6 arm of UPR to provide the active spliced XBP1 for sustaining UPR activation in the late phase.^583^

JEV infection requires Hsp70s in particular stages of its life cycle for survival and establishment of infection, including viral entry, replication,^194^ and maturation.^195^ Cell-surface Hsp70 directly interacts with JEV envelope protein.^192^ Antibodies against Hsp70 or 90 significantly block virus entry.^111^ It is also observed that Hsp70 colocalizes and directly interacts with JEV replicase complex. In addition, Hsp70 also interacts with NS3, NS5 and viral dsRNA that stabilizes VRC formation during JEV infection.^194,584^

### ER stress and antiviral

In mammalian cells, the ER stress is sensed and mediated by three ER transmembrane receptors: pancreatic ER kinase (PKR)-like ER kinase (PERK), inositol-requiring enzyme 1 (IRE1) and activating transcription factor 6 (ATF6). In resting cells, these three sensors are maintained in inactive states via interactions with the ER resident chaperone Grp78. When unfolded or misfolded proteins accumulate in the ER lumen, Grp78 dissociates from these three transducers, resulting in activation and initiation of the UPR.

Viruses also take advantage of or revolt ER stress response by different means for favour its life cycle at specific stage(s) of infection. The ER stress response constitutes a cellular process triggered by a variety of conditions disturbing protein folding in the ER. Eukaryotic cells have developed an evolutionarily conserved adaptive mechanism, i.e., the ER stress and UPR, aiming to clear unfolded/misfolded, and excessive amount of protein production; thereby restoring ER homeostasis. In cases ER stress cannot be resolved, UPR would be triggered and lead to cell death. ER stress and UPR could be observed in a large amount of virus infections, as most of viruses require host ER machinery for viral protein synthesis and modification. Virus infection usually causes ER stress. When large amount of misfolded/unfolded proteins or excessively expressed viral proteins accumulate inside ER lumen, ER stress response is triggered as indicated by fast elevated expression of ER chaperone proteins, including Grp78/Bip, Grp94, calnexin and calreticulin. It is well documented that ER stress response is triggered by a number of viruses, including influenza virus, infectious pancreatic necrosis virus (IPNV), Tula virus (TULV), PEDV, Bovine viral diarrhea virus (BVDV), DENV, JEV, HBV, HCV, Hepatitis E virus (HEV), HSV-1, canine distemper virus (CDV), RSV, simian virus 5 (SV5), CVB3, and HIV-1, etc.

In some cases, ER stress plays a role in virus pathogenesis. For instance, JEV, BVDV, TULV, severe acute respiratory syndrome coronavirus (SARS-CoV) and WNV have all been shown to induce apoptosis through UPR.^585–589^ Oxidative stress is mediated by ER stress during HCV infection.^21^ Certain viruses modulate ER stress response or preferentially activate different pathways of UPR. HCV infection activates the ATF6 pathway while blocks the IRE1 pathway. Instead, HBV infection stimulates both ATF6 and IRE1 signalling but has no effects on PERK signalling although both virus infect the same kind of hepatocytes.^590–592^ HSV-1 develops a virulence factor enabling dephosphorylation of eIF2α, one of the downstream effectors of the PERK pathway.^593^

Some consequences of UPR are beneficial for viral life cycle. For example, ATF6-induced expression of chaperone proteins may help viral protein folding and prevent protein aggregation. The PERK-eIF2α-activated ATF4 may help re-establish cell metabolism and resume protein translation. The IRE1-XBP1 pathway may facilitate virus replication by enhancing ER protein-folding ability and ER membrane biosynthesis.^594^ The activated ATF6 promotes the replication of ASFV and Lymphocytic choromeningitis virus (LCMV).^595,596^ DENV envelope protein directly interacts with Grp78, which provides a scaffold for its association with two other chaperones calnexin and calreticulin. This complex significantly improves the proper folding and stability of DENV E protein, thereby leading to increase of virus production.^597^ Other than DENV, Grp78 is also demonstrated to promote HCMV, JEV and RGNNV infections.^195,598,599^ The IRE1-XBP1 pathway is activated by IAV to facilitate its replication.^600^ However, other UPR outcomes are detrimental for virus replication. The PERK-eIF2α-mediated global translation attenuation is known as an antiviral response to restrict viral replication, such as infection by DENV or WNV.^583,601^ The activated PKR phosphorylates eIF2α at the ribosomal interface, which in turn causes a general inhibition of protein synthesis and blockage of VSV replication.^602^ The IRE1-XBP1(s)-mediated ER-associated protein degradation (ERAD) pathway reduces intracellular HBV particles by degrading its envelope proteins.^603^

Another approach of ER stress contributing to virus pathogenesis goes through modulating host cell immune responses. VSV, HCV and SARS-CoV are able to inhibit the type I IFN signaling pathway by activating the PERK signalling, which leads to the phosphorylation-dependent ubiquitination and subsequent degradation of IFNAR1, thereby promoting immune evasion and virus pathogenesis.^604,605^ WNV has also been reported to induce ER stress and inhibit type I IFN signaling pathway for escaping from the host immune response.^601^ US11 protein of HCMV activates UPR to facilitate the degradation of class I major histocompatibility complex (MHC1), leading to immune evasion.^606^ Moreover, ER stress is responsible for viral pathogenesis by interconnecting with the inflammatory responses. For example, HCV induces inflammatory responses by activating IRE1, which interacts with TRAF2 to phosphorylate JNK, leading to activation of inflammation mediators.^607^ NS4B and NS5A proteins of HCV activate NF-κB via ER stress-elicited calcium depletion and ROS production.^604^ The X protein (HBx) of HBV induces the expression of cyclo-oxygenase 2 (COS2), a key mediator of inflammation, through PERK-eIF2α-activated ATF4.^608^

### RNA chaperons and human diseases caused by viral infections

hnRNPs impact mRNA metabolism including transcript synthesis, processing and degradation as well as translation.^432^ The function of hnRNPs is determined or modulated by cellular localization. The mechanisms that regulate the nucleo-cytoplasmic shuttling are, therefore, of extreme importance. Most hnRNPs possess a conventional nuclear localization signal (NLS) and are predominantly present in the nucleus during steady state. They are able to translocate in the cytosol upon post-translational stimulation or by the recruitment of other hnRNPs.^609^ Post-translational modifications like methylation, phosphorylation, ubiquitination and sumoylation are reported to affect biological activity and subcellular localization of hnRNPs.^610^ As stated in the above sections, virus always employs different strategies to redistribute hnRNPs in host cells.^430,431,433,434^

How dysregulation of hnRNPs causing human diseases has gained an increasing interest. The expression level of hnRNPs is altered in many types of diseases, including varieties of human cancers and neurodegenerative diseases (e.g., spinal muscular atrophy (SMA), amyotrophic lateral sclerosis (ALS), Alzheimer’s disease (AD), and fronto-temporal lobe dementia). In this section, we mainly focus on the relationship between hnRNPs and diseases caused by virus infections.

Enteroviruses are common pathogens that cause human diseases worldwide. Although most enteroviral infections are subclinical, they may cause a wide spectrum of diseases including mild upper respiratory illness (common cold), febrile rash (hand, foot, and mouth disease and herpangina), aseptic meningitis, heart failture, pleurodynia, encephalitis, acute flaccid paralysis (paralytic poliomyelitis) and neonatal sepsis-like disease.^293,611^ Besides, several studies showed that enterovirus sequences could be detected in neuronal cell bodies of the spinal cord of 60–88% amyotrophic lateral sclerosis (ALS) patients, suggesting that persistent enterovirus infection may have a relationship with ALS.^295,297,299^ Enterovirus infections are supposed to cause or increase the risk of ALS; because the replication and translation of poliovirus, EV-A71, and coxsackievirus redistribute numerous hnRNPs (such as hnRNP A1) into the cytoplasm, the same localization during ALS pathogenesis. For example, hnRNP K, hnRNP A1, hnRNP M and hnRNP D shuttle into cytoplasm to assist virus replication or translation.^300,455,472,612–614^ Dislocated hnRNP A1 and loss of splicing function have been regarded as a toxic mechanism in ALS pathogenesis.^301^

HSV infection is one of the most common causes of infectious disease in humans.^302^ HSV infection often causes watery blisters in the skin or mucous membranes of the mouth, lips, nose, or genitals.^615^ As neurotropic and neuroinvasive viruses, HSV-1 and -2 persist in the infected individuals through hiding in the neuron cells from immune surveillance. When the carrier’s immunity compromised, HSV is reactivated that causes new sores. More seriously, accumulating evidence shows that HSV infection is associated with the pathogenesis of Alzheimer’s disease.^105,616,617^ During HSV infection, hnRNP K positively promotes HSV replication;^618^ while hnRNP A2/B1 negatively regulates HSV replication by triggering immunity response.^512^

HBV, HCV, EBV, HPV and HIV are oncogenic viruses that account for over 20% of human cancers. HCV or HBV infection leads to a wide spectrum of liver disease ranging from acute hepatitis (including fulminant hepatic failure) to chronic hepatitis, cirrhosis, and hepatocellular carcinoma (HCC).^619,620^ HDV can only infect people who are already infected with HBV. The coinfection of HDV and HBV increases the risk of liver cirrhosis and liver cancer.^621,622^ Except for damaging the liver, 10% of HBV infected patients also have other symptoms outside the liver, such as serum-sickness–like syndrome, membranous glomerulonephritis, acute necrotizing vasculitis (polyarteritis nodosa) and papular acrodermatitis of childhood (Gianotti–Crosti syndrome).^623^ Multiple steps of HCV reproduction need the presence of hnRNPs. The RNA replication of HCV needs the presence of hnRNP I, hnRNP C;^437,442,446^ while virus RNA translation requires hnRNP A1, hnRNP D, hnRNP I, hnRNP K and hnRNP L.^452,460,462–465^ Besides, hnRNP A2, hnRNP L, hnRNP U and hnRNP K are highly expressed in HCC tissue. They promote liver cancer progression.^8,304,305,624^

EBV has been implicated in several diseases, including infectious mononucleosis,^625^ Burkitt’s lymphoma,^626^ Hodgkin’s lymphoma,^627^ nasopharyngeal carcinoma,^628^ multiple sclerosis^629^ and lymphomatoid granulomatosis.^630^ Other diseases associated with EBV infections include Gianotti–Crosti syndrome, erythema multiforme, acute genital ulcers, oral hairy leukoplakia,^631^ and disorders related to α-synuclein aggregation (e.g. multiple system atrophy, dementia with Lewy bodies, and Parkinson’s disease).^632^ During EBV infection, hnRNP A1 and hnRNP C assist the polyadenylation of EBV RNA. hnRNP D takes part in cellular transformation-induced by EBV.^510^

HPVs are a group of DNA viruses with more than 150 members that infect cutaneous and mucosal epithelia. The acute infection of HPV causes benign cutaneous lesions such as non-genital and genital warts, or flat cervical condylomas.^633^ About 15 HPV subtypes that infect genital tract are capable of inducing malignant tumors, most commonly in the cervix.^308,309^ These cancer-associated HPVs are grouped into high-risk types, while those not associated with cervical cancer are regarded as low-risk types.^307^ Most infections by low-risk type HPVs are asymptomatic, except for infection by HPV6 and HPV11 that cause most cases of genital warts (condyloma acuminatum).^309^ HPV-related cancers are the result of long-term persistent infection with high-risk types. HPV16 and HPV18 are the most common carcinogenic types which account for a larger proportion of cervical cancers, squamous cell cancers and adenocarcinomas in all regions.^311^ HPVs are also implicated in the development of a variable proportion of vulvar, vaginal, penile, and oropharyngeal cancers, anal cancer,^634^ head and neck cancers,^635–637^ lung cancer and skin cancers. hnRNP A1 is involved in splicing of HPV RNA; while hnRNP H plays a role in the polyadenylation of HPV. hnRNP K and hnRNP I are required for viral protein translation.

Besides acquired immunodeficiency syndrome (AIDS), HIV infection is also able to cause human cancers because HIV infection impairs immunity system. People with AIDS are not only more easily infected with bacteria, viruses, fungi, and parasites;^638^ but at high risk for developing various viral-induced cancers as well, including Burkitt’s lymphoma, Kaposi’s sarcoma, primary central nervous system lymphoma, and cervical cancer.^639^ hnRNP A1, hnRNP D, hnRNP H regulate the splicing of HIV RNA.^538,548,550,551,556,563^ hnRNP K assists HIV transcription. And some other hnRNPs participate in HIV life cycle, including hnRNP A2, HnRNP A/B, hnRNP A2B1, hnRNP U, hnRNP C, hnRNP I and hnRNP K.^515,532–537,544–559,564^

### Potential therapeutic value by targeting stress proteins

Interest in targeting stress proteins in various diseases is increasing overtime. In this part, we will discuss the potential therapeutic value by targeting stress proteins.

First, targeting stress proteins have a wide spectrum of antiviral ability. For example, Hsp90 inhibitors, which are originally developed for anticancer, have been demonstrated to possess antiviral activity in cultured cells against poliovirus, rhinovirus, EV-A71,^107,109^ coxsackievirus,^124^ RSV,^112,113^ VSV, paramyxoviruses (HPIV2, HPIV3, SV5, SV41), influenza virus,^126^ CHIKV,^114^ HCV,^115,124^ and HSV,^131,144,145,148,149^ HBV,^135,169^ EBV,^146,147,163,164^ HCMV^151,152^ and HTLV.^153,185^ Depleting Hsp60 by siRNA functionally suppresses infections by influenza virus,^38,39^ DENV,^319^ HBV,^325,329,331^ HCV,^321,322^ Rotavirus,^323^ and HIV.^338^ Targeting hnRNP A1 is able to inhibit the reproduction of HIV,^539–541,563^ HPV,^503–505^ HCV,^471^ EV-A71^472^ and HTLV-1.^562^ Inhibition of hnRNP C could be a strategy to combat viral infections by HCV,^438^ poliovirus,^447,448^ DENV,^479–482^ EBV^501^ and HIV^557,558^ reproduction. Targeting HnRNP D blocks HCV and HIV replication.^452–454,548,556^ Dysfunction of hnRNP H effectively suppresses infections by EBV,^499,500^ HIV^538,550–552^ or RSV.^485–487^ Targeting PTB/hnRNP I is able to impair the reproduction of HCV,^442,460^ picornavirus,^89,456,459^ HPV^507^ and HIV^557,558^ Depriving of hnRNP K inhibits both DNA and RNA viruses’ reproduction, including influenza virus,^449,450^ EV-A71,^455^ DENV,^476^ VSV,^479–482^ HCMV,^488^ HBV,^490^ HSV,^492,493^ HPV,^508^ HIV^514,536,537^ and KSHV.^560,561^ hnRNP L and hnRNP M are important to HCV and enterovirus replication.^461–463,475^ Therefore, stress proteins are particularly attractive as antivirals targets for those lacking therapies and newly emerging viral diseases.

Second, under disease situations, the cellular need of stress proteins is usually stronger than that in normal conditions which enable specific selectivity of those drugs targeting stress proteins. For example, mutant p53 relies much more on Hsp90 function than wild type p53. Hsp90 inhibitor GM can easily disturb the association of mutant p53 with Hsp90, resulting in mutant p53 degradation while not affecting wild type p53.^310^ Additionally, stress proteins derived from stressed cells display higher affinity to clients and inhibitors. Tumor cell-derived Hsp90 exhibits a 100-fold higher binding affinity for 17-AAG than that from normal cells, since tumor cell-derived Hsp90 complexes with activating co-chaperones p23 and HOP exhibit increased ATPase activity and possess higher affinity to Hsp90 inhibitors. In contrast, Hsp90 in normal cells exists as an uncomplexed species with low ATPase activity and low affinity to Hsp90 inhibitors.^294^ Further study demonstrates that the difference is caused by a distinctive portion of Hsp90 forming complexes in cancer cells with oncogenic partners, such as Bcr-Abl-Hsp90 complex in mutant B-RAF-Hsp90 in SkMel28 melanoma cells, K562 chronic myeloid leukemia cells, Her3-Hsp90 and Raf1-Hsp90 complex in MDA-MB-468 breast cancer cells. Hsp90 inhibitor PU-H71 selectively binds to specific Hsp90-oncoprotein networks in these cancer cells.^570^ These characters of cancer cells enable stress proteins to exhibit stronger biological activity and to be discriminated by their inhibition under stress as compared with normal conditions.

Similarly, inhibitors of stress proteins show high prospect for antiviral. Hsp70 inhibitor JC40 inhibits pan-flavivirus (DENV2 and DENV4) replication in MDDCs, with negligible toxicity to host cells.^640^ These experiments highlight the feasibility of using HSP inhibitors therapeutically in humans.

Third, it is not observed drug resistance using HSP inhibitors for antiviral. For instance, Hsp90 inhibitors are refractory to develop drug resistance. This is clearly demonstrated in RSV infection.^113^ When RSV is repeatedly treated with Hsp90 inhibitors, no drug resistance was observed either in extensive passaging of the virus in cultured cells or in mice undergoing long-term treatment with Hsp90 inhibitors. Similar result is also observed in DENV infections.^640^ Treatment of DENV up to 10 passages with Hsp70 inhibitor JC40 does not cause any drug resistance. The lack of viral drug resistance to Hsp90 and Hsp70 inhibitors suggests that such an antiviral approach may be particularly useful for treating chronic viral infections in which drug resistance is most frequently observed.

The three features of stress proteins inhibitors make them extremely powerful antiviral agents, suggesting great application potential for treating the diseases caused by virus infections, such as COVID-19.

### Challenges and perspectives: target-based drug development

#### *Targeting Hsp90 for antivirus drug development*

Hsp90 is thought to be the most abundant and evolutionarily conserved heat shock protein. A unique pocket in N-terminal region of Hsp90 is required for binding with ATP and co-chaperones.^641^ Hsp90 forms a flexible dimer by interaction of C-terminal domains. The formation and dissociation of compact dimers involving N- terminal domains are important for the molecular chaperone activity.^642^ The middle domain of Hsp90 tends to recruit and facilitate unfolded client proteins to assemble. The C-terminal domain of Hsp90 possesses a highly conserved peptide sequence for interacting with co-chaperones.^643^ Over 20 co-chaperones selectively interact with Hsp90 to regulate ATPase activity and recruit client proteins to assemble a big complex under certain conditions.^642–645^ It has been shown that more than 300 client proteins require Hsp90 and co-chaperones for folding and maturation.^646^ The mechanism of selectivity may rely on the direct interaction of co-chaperones with specific clients. Therefore, most Hsp90 inhibitors achieve their inhibitory effects by suppressing the ATPase activity or disrupting the interaction between Hsp90 and its co-chaperones.

Expression of Hsp90 and its client proteins are increased during viral infection and in most cancer cells. Hsp90 as an effective anti-cancer drug target has already grabbed attention; and a series of Hsp90 inhibitors as potential drugs have been intensively investigated in the laboratories, preclinical and clinical scenarios. The successful use of Hsp90 inhibitors in cancer therapy makes it much easier to apply them for treating virus infections.

As described above, we have comprehensively summarized the functions of Hsp90 and its client proteins in a diversity of virus reproduction processes. The potential of Hsp90 inhibitors has been well demonstrated to protect cultured cells against infections by EV-A71,^107,109^ poliovirus, rhinovirus, coxsackievirus,^124^ paramyxoviruses (HPIV2, HPIV3, SV5, SV41),VSV, RSV,^112,113^ influenza virus,^126^ CHIKV,^114^ HCV,^115,124^ HSV,^131,144,145,148,149^ HBV,^135,169^ EBV,^146,147,163,164^ HCMV,^151,152^ and HTLV.^153,185^ Notably, administration of Hsp90 inhibitors to infected animals exhibits little toxicity while potently suppresses the replication of poliovirus,^107,109^ EBV,^163,164^ HBV,^135^ CHIKV^114^ and HCV.^647^ These experiments highlight the feasibility of using these inhibitors therapeutically in clinic.

Here we would briefly enumerate the present Hsp90 inhibitors and their potential for antiviral therapies. Most Hsp90 inhibitors hamper Hsp90 function by competitively binding to the ATP binding site of HSP90, blocking the interaction with co-chaperones, or modulating acetylation. By doing so, we also try to address the possibility of repurposing Hsp90 inhibitors as candidate antiviral drugs (Table 2).

**Table 2:** Hsp90 inhibitors

Inhibition type	Subtype	Inhibitor and references
Targeting Hsp90 ATPase Activity	Ansamycins	Geldanamycin (GM)^649^
		Tanespimycin (17-AAG)^650^
		Alvespimycin (17-DMAG)^651^
		Retaspimycin hydrochloride (IPI-504)^652^
	Non-ansamycins	Luminespib (AUY922)^653^
		Ganetespib (STA-9090)
		BIIB021
		Onalespib (AT13387)^656^
		SNX-5422 (PF-04929113)
	Blocking Hsp90 C-terminal ATPase activity	Novobiocin^658,659^
		Deguelin^660^
		Epigallocatechin gallate (ECGC)^662^
Disrupting Hsp90 and Its Co-chaperones	Targeting Hsp90-Cdc37 complex	Celastrol^663–667^
		Aferin A
		Sulforaphane
		Kongensin A
		Platycodin D
		Pep-1^668^
	Targeting Hsp90-Hop-Hsp70 complex	Six active compounds^669^
Blocking Deacetylation of Hsp90		Vorinostat (SAHA)^670^
		LAQ824^671^
		Romidepsin^672^
Hsp90 cleavage	Enzymatic cleavage	Histone deacetylase inhibitors^675^
		Proteasome inhibitors^676^
	Non-enzymatic cleavage	Ascorbate/Menadione^674^
		Oxidative stress (H2O2)

#### *Inhibitors targeting Hsp90 ATPase activity*

Some Hsp90 inhibitors competitively bind to the ATP pocket in the N-terminus of Hsp90, leading to blocking of ATP hydrolysis and the closure of N-terminus of Hsp90 dimer. In addition, another ATP binding site has been found in the C-terminus of Hsp90.^648^ Recently, some natural products and their derivatives have been reported to competitively bind to the ATP pocket in the C-terminus of Hsp90. Generally, inhibitors of Hsp90 ATPase are classified into three types: (i) ansamycins, (ii) non-ansamycins and (iii) those block Hsp90 C-terminal ATPase activity. Ansamycins are antibiotics that possess the benzoquinone as structure core. These antibiotics include geldanamycin (GA), herbimycin A, and the macbecins. They inhibit Hsp90 activity and degrade its client proteins.^649^ GA competitively binds to the ATP pocket in the N-terminus and inhibits the ATPase activity.^649^ It has shown potent antiviral activity against coronavirus infection in the culture cells,^145^ indicating a good potential for treating COVID-19. Tanespimycin (17-allylamino-17-demethoxygeldanamycin, 17-AAG) is an analogue of GA.^650,651^ Alvespimycin (17-Dimethylaminoethylamino-17-demethoxygeldanamycin, 17-DMAG) is an analogue of 17-AAG, with high solubility in water. In addition, retaspimycin hydrochloride (IPI-504) is a more water-soluble derivative of 17-AAG.^652^ Non-ansamycin inhibitors of Hsp90 include Luminespib,^653^ BIIB021,^654^ Ganetespib,^655^ Onalespib,^656^ SNX-5422,^657^ etc.

Another type of Hsp90 inhibitors binds to the C-terminal ATP pocket rather than N-terminal ATP pocket. Novobiocin blocks the C-terminal ATPase activity.^658,659^ In in vitro and in vivo assays, treatment with novobiocin strongly reduces the expression of Hsp90-dependent client proteins, such as Raf-1 and p60v-src. A natural rotenoid, deguelin, is suggested to bind to the ATP pocket in the C-terminus without affecting the ATP pocket in the N-terminus.^660^ Treating with deguelin leads to reduced Hsp90 clients, such as Cdk4, Akt and MEK1/2.^661^ Like deguelin, epigallocatechin gallate (ECGC) is reported to bind to the ATP pocket in the C-terminus of Hsp90 without affecting the N-terminal ATP pocket.^662^ Compared with inhibition of the N-terminal ATP pocket of Hsp90, inhibitors targeting the C-terminal ATP pocket lead to a stability of Hsp70 and a negative instability of client proteins of Hsp90. However, the potential of these C-terminal inhibitors and their molecular mechanisms remain elusive. The structural features of the C-terminal domain of Hsp90 need to be further analysed. Such analysis may provide important hints on how to design effective Hsp90 inhibitors without HSR induction.

#### *Inhibitors of Hsp90’s Co-chaperones*

As the functions of the Hsp90 chaperone machinery are highly dependent on the associated co-chaperones, it is possible to selectively inhibit downstream signaling of Hsp90 by disrupting certain protein–protein interactions. Inhibitors targeting the interactions may present an alternative approach to prevent the toxicity induced by other Hsp90 inhibitors. As a result, great efforts are put into disrupting the interaction between Hsp90 and its co-chaperones and are rewarded recently in this area. Here we briefly present the advance in Hsp90-Cdc37 complex and Hsp90-Hop-Hsp70 ternary complex.

Human Cdc37 is a well-studied co-chaperone of Hsp90. The middle domain and C-terminus of Cdc37 are critical for the interaction with Hsp90. Currently, most of the reported agents for manipulating the Hsp90-Cdc37 interaction are natural products and their derivatives. These agents include celastrol, aferin A, sulforaphane, kongensin A and platycodin D. They are capable of dissociating the Hsp90-Cdc37 complex.^663–667^ In addition, a peptide (Pep-1, Ac-KHFGMLRRWDD-NH2) is developed and shows strong inhibitory effects on the formation of Hsp90-Cdc37 complex.^668^ Another well-known Hsp90/co-chaperone complex is Hsp90-Hop-Hsp70 ternary complex, which facilitates the transfer of unfolded client proteins from Hsp70 to Hsp90. Notably, six active compounds have been identified after screening the NCGC compound library.^669^ These compounds possess similar structural cores and have no effect on Hsp70 expression.

Studies on Hsp90-co-chaperone inhibitors are limited. Great effort should be paid on the efficacy and toxicity for further drug design and clinical studies.

#### *Hsp90 inhibitors blocking deacetylation of Hsp90*

The chaperone function of Hsp90 is also controlled by posttranslational modification (PTM). The well-studied PTMs in Hsp90 are acetylation and deacetylation in the M-domain of Hsp90. Vorinostat (suberoylanilide hydroxamic acid, SAHA) dissociates Her2/ErbB2 from Hsp90 via acetylation of Hsp90 that leads to degradation of Her2/ErbB2.^670^ LAQ824 induces Hsp90 acetylation that reduces Hsp90 client protein levels.^671^ Romidepsin is involved in the dissociation of mutant p53 and Raf-1 from Hsp90 via acetylation of Hsp90.^672^ In addition, the nuclear import of IAV polymerase needs the deacetylation of Hsp90 which is strictly regulated by histone deacetylases 6/8 (HDAC6/8).^120^ HDAC6/8 inhibitors efficiently limit polymerase nuclear import and suppress virus replication.^120^ Therefore, Hsp90 inhibitors that block Hsp90 deacetylation would potentially be used to treat influenza A virus infection. Their potential usage for combating other viruses (such as SARS-CoV-2) needs to be extensively explored.

#### *Novel class of Hsp90 inhibitors for Hsp90 cleavage*

Recently, Hsp90 cleavage has been observed under various stimuli such as UV irradiation,^673^ ascorbate/menadione,^674^ HDAC inhibitors,^675^ proteasome inhibitors^676^ etc. Therefore, Hsp90 cleavage is considered as another mechanism. Hsp90 cleavage induced by these inhibitors can be classified into two types: enzymatic cleavage and non-enzymatic cleavage.^674,676^ The enzymatic cleavage produces a 55 kDa fragment of Hsp90 via caspase 10 activation, while the non-enzymatic cleavage utilizes reactive oxygen species (ROS) to chemically degrade Hsp90 to an ~70 kDa fragment. Additionally, some substances have been reported to present Hsp90 cleavage activity, but the mechanism remains unclear.

##### Targeting Hsp70s for drug development

One kind of antiviral drugs are currently designed based on different properties of HSPs. Considering Hsp70 and Hsc70 are potential targets for HCV, they load the DCs with Hsp70 for a prolonged potent antigen- and tumor-specific T cell responses directed against multiple epitopes.^677^ Hsp70 inhibitors (such as quercetin, VER155008 and JC40) also show great potential for treating Flavivirus and Enterovirus infections.^202,208,640^ Inhibitors blocking the interaction between Grp78 and spike protein of coronavirus would be a useful approach for combating the infection by SARS-CoV, MERS-CoV, and SARS-CoV-2 infections.^196,387,571^ However, the efficacy and toxicity of these inhibitors are not yet well investigated in clinic studies.

##### Targeting Hsp60s for drug development

As described above, Hsp60s play critical roles in different diseases including autoimmune diseases, human cancers and virus infection-induced diseases. Studies show that silencing of Hsp60s by siRNA significantly decrease influenza virus,^38,39^ DENV^319^ and HBV^325,331^ reproduction by activating immunity response; and is capable of releasing HCV^321,322^, Rotavirus,^323^ HBV^329^ and HIV^338^ infection-induced pathogenesis. Therefore, small molecule regents targeting Hsp60s are potentially useful as therapeutics in these diseases.^678,679^ Currently, the known Hsp60 inhibitors are either from natural products or synthetic compounds (Table 3). Mechanistically, they can be grouped as two types. Type I inhibitors block ATP binding and hydrolysis. Type II inhibitors covalently interact with certain cysteine residues of Hsp60. However, the detailed binding sites have not been identified. The natural products of Hsp60 inhibitors include mizoribine, epolactaene, myrtucommulone A, Stephacidin B. Mizoribine is an imidazole nucleoside antibiotics isolated from *Eupenicillium brefeldianum.*^680^ It directly binds to Hsp60 and inhibits the chaperone activity of the Hsp60-Hsp10 complex.^33^ Epolactaene is isolated from the fungal strain *Penicillium* sp. BM 1689-P.^681^ Its derivate *tert*-butyl ester ETB has similar activity as epolactaene.^682^ They inhibit Hsp60-Hsp10’s chaperoning activity by covalent and selective bound to Hsp60 at residue of Cys442, close to the ATP binding pocket.^683,684^ Interestingly, ETB does not inhibit the ATPase activity of Hsp60,^685^ suggesting that the covalent interaction between ETB and Cys442 may allosterically modulate Hsp60-Hsp10’s chaperoning activity without interfering with its ATPase activity. Myrtucommulone A (MC) binds to Hsp60 and inhibits the refolding activity of the Hsp60-Hsp10 complex.^686^ MC is a non-prenylated acylphloroglucinol with multiple reported bioactivities, including antibacterial,^687,688^ antioxidant,^689^ anti-inflammatory,^690,691^ and anti-tumor properties.^692,693^ In addition to these natural products, several other natural products are also reported to interact with Hsp60 without direct proof. Stephacidin B is isolated from *Aspergillus ochraceus* WC76466 while Avrainvillamide is isolated from *Aspergillus* sp. CNC358.^694,695^ Interestingly, dimeric stephacidin B is converted into monomeric avrainvillamide in tissue culture media which implies avrainvillamide is the actual active species.^696^ Pull down assay only shows Hsp60 may be a putative target.^696^ Further validation studies have yet to be performed.

**Table 3:** Hsp60 inhibitors

Types	Inhibitors	Mechanism and references
Natural Products	Mizoribine	To inhibit chaperone activity of the Hsp60-Hsp10 complex; to block ATP binding and hydrolysis^33^
	Epolactaene	To inhibit chaperone activity of the Hsp60-Hsp10 complex^683^
	Myrtucommulone A (MC)	To inhibit chaperone activity of the Hsp60-Hsp10 complex^686^
	Stephacidin B	Undetermined^694^
	Avrainvillamide	Undetermined^695^
Synthetic Compounds	*O*-carboranyl-phenoxyacetanilide	To inhibit chaperone activity of the Hsp60-Hsp10 complex; to block ATP binding and hydrolysis^685^
	Gold porphyrins	To inhibit chaperone activity of the Hsp60-Hsp10 complex

Besides the natural products identified above as potential Hsp60 inhibitors, quite a few synthetic molecules have also been discovered to be able to modulate Hsp60 activity. *O*-carboranyl-phenoxyacetanilide is firstly identified as an HIF-1α inhibitor.^697^ Later it is found that *O*-carboranylphenoxyacetanilide selectively and directly binds to Hsp60 and inhibits Hsp60-Hsp10’s ATPase activity and refolding activity.^685^ Hsp60 can interact with HIF-1α, suggesting that inhibition of HIF-1α activity by carboranylphenoxyacetanilide is possibly through targeting Hsp60.^685^ The other class of synthetic compounds identified to inhibit Hsp60 is gold porphyrins.^698^ It directly binds to Hsp60 and inhibits the refolding activity of the Hsp60-Hsp10 complex.

Although several Hsp60 inhibitors potently suppress virus reproduction, a lot of work remains to be done to verify if they can serve as drugs for treating diseases caused by virus infections.

#### *Targeting hnRNPs for antivirus drug development*

Drugs targeting RNA chaperones may have a wide spectrum of antivirus capability. hnRNP A1 participates the reproduction of HIV,^539–541,563^ HPV,^503–505^ HCV,^471^ EV-A71^472^ and HTLV-1.^562^ Inhibition of hnRNP C suppresses the replication of HCV,^438^ poliovirus,^447,448^ DENV,^479–482^ EBV,^501^ and HIV.^557,558^ hnRNP D is required for HCV^452–454^ and HIV^548,556^ replication. HnRNP H is involved in multiplication of EBV,^499,500^ HIV,^538,550–552^ and RSV^485–487^ infections. PTB/hnRNP I depletion is able to impair the life cycle of HCV,^442,460^ picornavirus,^89,456,459^ HPV^507^ and HIV.^557,558^ Depriving of hnRNP K reduces the reproduction of influenza,^449,450^ EV-A71,^455^ DENV^476^ VSV,^479–482^ HCMV,^488^ HBV,^490^ HSV,^492,493^ HPV,^508^ HIV,^514,536,537^ and KSHV.^560,561^ HnRNP L and hnRNP M participate in HCV and enterovirus replication.^461–463,475^ Of note, the relationship between HIV and RNA chaperones is very strong. Most hnRNPs participates in HIV life cycle, including hnRNPA1,^563^ hnRNPA2,^553–555^ hnRNP A/B,^544,545,547^ hnRNP A2B1,^557,558^ hnRNP U,^559^ hnRNP C, hnRNP D,^548,556^ hnRNP H,^538,550,551^ hnRNP I^557,558,564^ and hnRNP K.^514,536,537^

However, the available regents developed targeting hnRNPs as antiviral drugs are limited. To date, only three agents targeting hnRNPs have been reported. Apigenin is a flavonoid abundant in fruits and vegetables. It targets hnRNP A2 and blocks hnRNP A2 dimerization thereafter affects the alternative splicing of key mRNAs.^699^ Apigenin efficiently inhibits the interaction of hnRNP A1 and A2 with EV-A71 RNA thereby inhibits EV-A71 RNA translation.^472^ Quercetin is also a flavonoid abundantly present in plants. It binds to the C-terminal region of hnRNP A1, impairing the ability of hnRNP A1 to shuttle between the nucleus and cytoplasm and ultimately resulting in its cytoplasmic retention.^700^ In addition, Quercetin down-regulates hnRNP A1 expression.^701^ The antiviral ability of Quercetin has been verified in some viruses such as inhibiting influenza entry,^702^ EV-A71,^703^ and HCV,^208^ JEV and DENV reproduction.^704^ However, in these researches, they explain the antivirus mechanism of Quercetin is by inhibiting Hsp70 expression^208^ or inhibiting virus proteases^703^ without mentioning the function of hnRNP A1. Another compound VPC-80051 targets RNA binding motif of hnRNP A1 and alters hnRNP A1 splicing activity.^705^ Its antiviral ability remains to be further investigated.

##### Targeting ER stress pathways for drug development

During the past few years, the enlarging role of ER stress-mediated pathways is becoming quite an attractive topic for broad-spectrum antiviral therapy, especially in the field of virus reproduction and pathogenesis, considerable progress has also been made for potential antiviral agents development.

Among pathways involved in ER stress, the three UPR pathways are the most thoroughly studied, thus antiviral targets related with these pathways are considered as the first class. Extensive investigation for antiviral development has been put int to the PERK-eIF2α pathway.

A small chemical compound named salubrinal, identified by Boyce and his colleagues, specifically inhibits the formation of PP1/GADD34 complex as well as suppresses HSV γ134.5-mediated eIF2α dephosphorylation. It could also significantly reduce HSV replication.^706^ Their research suggested that Salubrinal was able to attenuate PP1/GADD34-dependent eIF2α dephosphorylation, which would in turn inhibit DENV replication.^707^ A glucose analog 2-Deoxy-D-Glucose is responsible for the activation and phosphorylation of eIF2α, leading to suppression of herpesvirus (KSHV) replication. This agent is a potential candidate for anti-herpesvirus, and hopefully is tested in clinical therapies.^708^

Other than eIF2α, tremendous efforts have also been made in developing drugs to target IRE1-XBP1 pathway. 3,5-dibromosalicyladehyde is an endoribonuclease that specifically interacts with IRE1 and blocks the downstream IAV replication.^600^ WP1130 is another IRE1-XBP1 inhibitor with a broad-spectrum antiviral effects against multiple viruses, namely MNV-1, LCV and so on.^709^

ER stress-mediated apoptosis signaling is another noteworthy pathway for drug development and selection. Rana catesbeiana ribonuclease (RC-RNase) is reported to be efficient in suppression of JEV replication via activation of caspase-3, caspase-8 and caspase-9 pathways.^710^ Dubble-stranded RNA activated caspase oligomerizer (DRACO) is reported to have broad anti-viral effects in clinical therapy. DRACO selectively activates apoptosis in cells infected with dsDNA viruses but has no harmful effect on normal healthy cells. Over 15 different kind of viruses can be eradicated by DRACO, including HIV and Dengue Virus.^711^ Drugs targeting other signal transducers are also investigated, for example JNK, JAK, Bcl-2 and CHOP. A promising agent Vaticanol B can protect host cells from ER stress-induced apoptosis.^712,713^

Some researchers choose to focus on viral proteins that could trigger ER-stress, including non- envelope glycoproteins of SFV, precursor glycoprotein of LCMV, glycoproteins TULV, several non-structural protein of SARS-CoV, multiple non-structural proteins of flavivirus family, envelope protein of PEDV, E1, E2 and non-structral protein of HCV, surface protein of MHV, ICP0 glycoprotein B of HSV1, structural protein 4 of CHIKV. Viral proteins that are responsible for UPR activation and apoptosis induction are given special attentions such as HCV (E1, E2, core), HCMV (pUL37x1, pUL38) and SV5 V protein. Great advances have been made in this area, current progress includes, Clemizol for HCV NS4B, vaccine vectors for HSV-1 γ134, and Norakin for IAV hemagglutinin A.^714,715^

Due to their potential side effects on normal cell functions, the usage of inhibitors targeting stress proteins are very cautious in clinic settings; and the gone with wind infection manner of some virus (e.g., SARS-CoV) limits the opportunity of clinic studies, giving rise to additional challenges for developing stress protein-based drugs against the common and special infectious diseases such as severe acute respiratory symptom (SARS) caused by coronavirus.
